# AP-1γ2 is an adaptor protein 1 variant required for endosome-to-Golgi trafficking of the mannose-6-P receptor (CI-MPR) and ATP7B copper transporter

**DOI:** 10.1016/j.jbc.2024.105700

**Published:** 2024-02-01

**Authors:** Lucas Alves Tavares, Roger Luiz Rodrigues, Cristina Santos da Costa, Jonas Alburqueque Nascimento, Julianne Vargas de Carvalho, Andreia Nogueira de Carvalho, Gonzalo A. Mardones, Luis L.P. daSilva

**Affiliations:** 1 Center for Virology Research and Department of Cell and Molecular Biology, Ribeirão Preto Medical School, University of São Paulo, Ribeirão Preto, SP, Brazil; 2 Escuela de Medicina, Facultad de Medicina y Ciencia, Universidad San Sebastián, Valdivia, Chile

**Keywords:** AP-1γ2, clathrin, CI-MPR, ATP7B, retrograde transport, receptor recycling

## Abstract

Selective retrograde transport from endosomes back to the trans-Golgi network (TGN) is important for maintaining protein homeostasis, recycling receptors, and returning molecules that were transported to the wrong compartments. Two important transmembrane proteins directed to this pathway are the Cation-Independent Mannose-6-phosphate receptor (CI-MPR) and the ATP7B copper transporter. Among CI-MPR functions is the delivery of acid hydrolases to lysosomes, while ATP7B facilitates the transport of cytosolic copper ions into organelles or the extracellular space. Precise subcellular localization of CI-MPR and ATP7B is essential for the proper functioning of these proteins. This study shows that both CI-MPR and ATP7B interact with a variant of the clathrin adaptor 1 (AP-1) complex that contains a specific isoform of the γ-adaptin subunit called γ2. Through synchronized anterograde trafficking and cell-surface uptake assays, we demonstrated that AP-1γ2 is dispensable for ATP7B and CI-MPR exit from the TGN while being critically required for ATP7B and CI-MPR retrieval from endosomes to the TGN. Moreover, AP-1γ2 depletion leads to the retention of endocytosed CI-MPR in endosomes enriched in retromer complex subunits. These data underscore the importance of AP-1γ2 as a key component in the sorting and trafficking machinery of CI-MPR and ATP7B, highlighting its essential role in the transport of proteins from endosomes.

The sorting of transmembrane proteins in the late secretory pathway relies on physical interactions between targeting signals in their cytosolic domains and adaptor proteins found in transport vesicle coats. These interactions determine the efficiency and specificity of sorting, influencing transmembrane proteins' localization, processing, and function. Within this context, two types of transmembrane receptors, the Cation-Dependent Mannose-6-phosphate Receptor (CD-MPR) and the Cation-Independent MPR (CI-MPR), are responsible for delivering soluble lysosomal hydrolases tagged with Mannose-6-phosphate (M6P) residues from the trans-Golgi network (TGN) to early endosomes using clathrin-coated vesicles (CCVs) (1, 2, 3). The acidic environment of endosomes facilitates the dissociation of MPRs from their ligands (4, 5, 6), and the receptors are recycled back to the TGN for further rounds of transport (7, 8, 9, 10, 11, 12).

In addition to sorting hydrolases, CI-MPR can interact with non-M6P-containing ligands, such as insulin-like growth factor II (IGF-II), earning the alternative name of IGF-II receptor. As a result, CI-MPR serves other functions, including cell mobility, growth, and regulation of apoptosis (13, 14, 15). Therefore, CI-MPR is thought to follow a more intricate cellular pathway than CD-MPR, being directed from the TGN and endosomes to the plasma membrane, where it is subsequently endocytosed and eventually returned to endosomes and the TGN (7, 8, 9, 10, 11, 12).

Similarly, the P-type ATPase copper transporters ATP7A and ATP7B are critical in maintaining cellular copper homeostasis by shuttling between the TGN, endosomes, and the plasma membrane. Copper is an essential micronutrient necessary for the function of oxygen-dependent enzymes; however, an excessive accumulation of Cu+ can be toxic. ATP7A is expressed in various tissues, while ATP7B is primarily found in the liver, with lower expression levels in the brain and kidney (16). Under basal conditions, ATP7A and ATP7B are primarily localized in the TGN (17, 18). However, when intracellular copper levels rise, these transporters redistribute from the TGN to peripheral cytoplasmic vesicles and the cell surface, enabling them to efflux excess copper from the cytosol. Following decreased copper levels, ATP7A and ATP7B return to the TGN to maintain homeostasis (19, 20, 21, 22).

The intracellular trafficking of MPRs and ATP7A/B involves the clathrin adaptor protein complex 1 (AP-1) (23, 24). AP-1 belongs to a family of five heterotetrameric complexes (25, 26), and is potentially the most versatile AP with the largest number of subunit isoforms reported. These include two γ (γ1 and γ2), two μ1 (μ1A and μ1B), three σ1 (σ1A, σ1B, and σ1C), and a single β (β1) subunit that may form alternative complexes (27). The interaction of AP-1 with the cytosolic tail of transmembrane proteins occurs *via* at least two types of sorting signals (1): the dileucine-based motif ((D/E)xxxL(L/I/M)), which interacts with the hemicomplex composed by γ/σ1 subunits (28, 29, 30); and (2) the tyrosine-based motif (Y_XX_Ø), which interacts with the μ1 subunits (31, 32, 33).

AP-1 has initially been implicated in the packaging at the TGN of CI-MPR into CCVs destined for early endosomes (34, 35, 36, 37). Additionally, AP-1 was reported to mediate the retrograde trafficking of CI-MPR from the early endosomes to the TGN (37, 38, 39, 40, 41, 42). Furthermore, the proper retrograde transport of ATP7B from endosomes to the TGN relies on the intact dileucine motif present in the C-terminal tail of ATP7B (22, 43, 44, 45), and functional AP-1 (45, 46, 47). Notably, AP-1 physically interacts with ATP7B, which is crucial for retrieving ATP7B from endosomes to the TGN (44, 45, 47).

Importantly, these studies on the function of AP-1 in CI-MPR and ATP7B trafficking assessed only the roles of μ1A or γ1 subunits but not the γ2 subunit isoform. Previous studies demonstrated that γ2 may form an AP-1 complex variant (30, 48), herein termed AP-1γ2 (Fig. 1*A*), but knowledge is limited regarding the cellular roles played by this alternative AP-1 complex. Depletion of γ2, but not γ1, impairs the targeting of endocytosed Epidermal Growth Factor (EGF) Receptor-EGF complexes for degradation *via* the multivesicular body pathway (49, 50, 51). In addition, γ2 was shown to be required for HIV-1 Nef-mediated targeting of CD4 and MHC-I for lysosomal degradation (51, 52).

**Figure 1:**
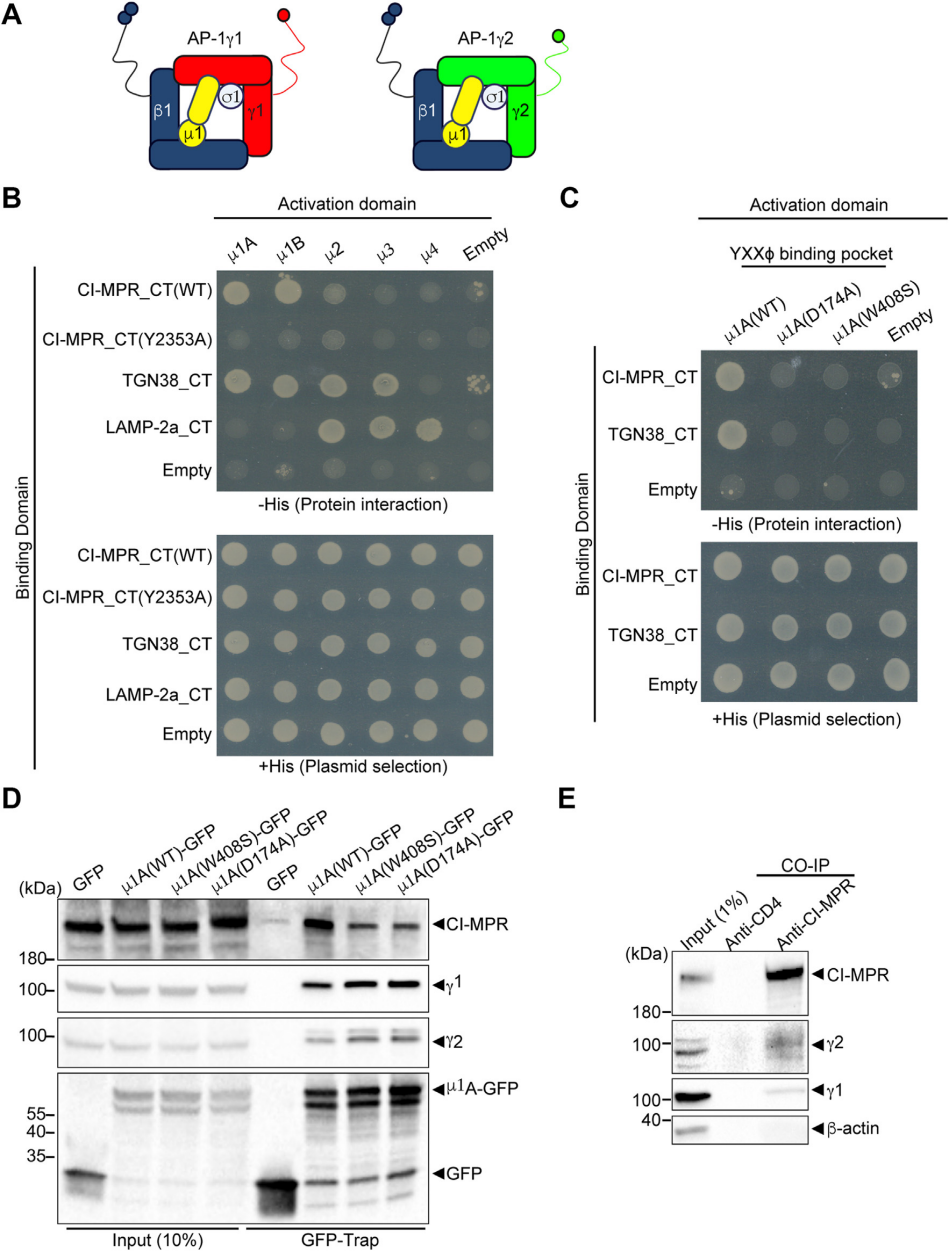
**CI-MPR interacts with both AP-1γ1 and AP-1γ2 complex variants.***A*, schematic representation of the AP-1 complex variants comprising either γ-adaptin isoforms, γ1 or γ2, which form complexes termed AP-1γ1 and AP-1γ2, respectively, together with the other three subunits: β1, μ1, and σ1. *B*, yeast two-hybrid assays of CI-MPR cytosolic tail (CI-MPR_CT) with the medium subunits (μ) of AP-1 to AP-4. Yeast growth in medium without histidine (-HIS) indicates interaction between the proteins tested. *C*, evaluation of CI-MPR interaction with μ1A containing two individual mutations in its tyrosine-binding pocket. Reduced yeast growth indicates that both μ1A mutants are defective in the interaction with CI-MPR_CT or TGN38_CT. *D*, GFP-Trap Co-IP assays using lysates of HEK cells expressing GFP, μ1A(WT)-GFP, μ1A(W408S)-GFP or μ1A(D174A)-GFP. CI-MPR co-IP with μ1A(WT)-GFP at higher levels compared to GFP, μ1A(W408S)-GFP or μ1A(D174A)-GFP. Input represents 5% of cell lysates used in the Co-IP assays. *E*, HeLa cell lysates were subjected to immunoprecipitation with anti-CD4 (negative control) or anti-CI-MPR antibodies. The samples were then processed for SDS-PAGE and Western blot for CI-MPR, γ1, γ2, and GAPDH. Input represents 1% of the samples.

Here, we show that AP-1γ2 interacts with CI-MPR and ATP7B, and is required for proper subcellular distribution of these transmembrane proteins. Although AP-1γ2 is dispensable for CI-MPR and ATP7B export from the TGN, the depletion of AP-1γ2 significantly disrupts the retrograde trafficking of CI-MPR and ATP7B from endosomes to the TGN, leading to an aberrant increase in cell surface localization. Specifically, in cells lacking AP-1γ2, an endocytosed CI-MPR-based reporter fails to reach the TGN and accumulates in endosomes enriched in retromer components. This observation leads us to suggest that AP-1γ2 is essential for the optimal functioning of retrograde transport carriers formed in early endosomes and destined to the TGN. Taken together, the data underscores the importance of AP-1γ2 as a key component in the sorting and trafficking machinery of CI-MPR and ATP7B, highlighting its essential role in the transport of proteins from endosomes.

## Results

### CI-MPR interacts with AP-1 complex variants containing either γ1 or γ2 subunits

The AP-1γ1 complex variant has been strongly associated with bidirectional CI-MPR trafficking between the TGN and endosomes (23), but the involvement of the AP-1γ2 variant remains at issue. To investigate the interaction of CI-MPR with both AP-1γ1 and AP-1γ2 variants (Fig. 1*A*), we first performed yeast two-hybrid assays using the CI-MPR cytosolic tail (CI-MPR_CT) and the μ subunit of different AP complexes. The cytosolic tail of TGN38 and Lamp2a served as positive controls for μ subunit interactions (53). We found a robust interaction of CI-MPR_CT with the μ1A subunit of AP-1 and its polarized epithelial cell–specific isoform μ1B (25). This interaction is disrupted by a Y/A substitution in the CI-MPR_CT tyrosine-based motif (_2353_YSKV_2356_/_2353_ASKV_2356_) (Fig. 1*B*). Consistent with previous findings, the μ1A tyrosine-binding pocket (54) is also essential for CI-MPR_CT binding (Fig. 1*C*). These data were confirmed by GFP-Trap co-IP assays showing that μ1A-GFP interacts with endogenous CI-MPR from cell lysates and that this interaction was compromised by mutating the μ1A tyrosine-binding pocket (Fig. 1*D*). Importantly, co-IP of endogenous γ1 and γ2 subunits indicate that μ1A-GFP incorporates into either AP-1γ1 or AP-1γ2 complex variants. As expected, incorporation was not prevented by the tyrosine-binding pocket mutation (Fig. 1*D*). To further investigate if CI-MPR binds the AP-1 variant containing γ2, we performed co-IP assays with the endogenous CI-MPR. The results show that both γ1 and γ2 precipitate with CI-MPR (Fig. 1*E*), indicating their ability to form complexes with this receptor.

### The γ2 adaptin is essential for normal CI-MPR intracellular distribution

Given that CI-MPR interacts with AP-1 complexes containing either γ1 or γ2, to confirm that μ1A serves as a shared subunit for both complexes, we investigated the behavior of γ1 and γ2-adaptins in HeLa μ1A CRISPR/Cas9 KO cells (55). Co-immunostaining of γ1 and γ2 in HeLa cells shows their partially distinct subcellular distribution (Fig. S1, *A* and *F*), confirming our previous observations (51, 52). Notably, the punctate patterns of γ1 or γ2 were lost in μ1A KO cells (Fig. S1*B*) but were rescued upon expression of either μ1A(WT)-GFP or tyrosine-binding pocket mutants of μ1A-GFP (Fig. S1, *C*–*F*). Western blot analysis of total cell extracts confirmed that μ1A depletion causes a strong decrease in the overall levels of γ1 and γ2, which are restored by the expression of μ1A(WT)-GFP (Fig. S1*G*). Therefore, the stability of both AP-1-γ1 and AP-1-γ2 complex variants depends on μ1A subunit expression, a phenotype previously reported (51, 52). Importantly, these results support that both γ1 and γ2-adaptins are components of AP-1 complex variants containing μ1A.

Since the stability of both γ1 and γ2 adaptins is dependent on μ1A subunit expression, we initially investigated CI-MPR trafficking in the context of γ1/γ2 co-depletion by using μ1A KO cells. In WT cells, CI-MPR localized mainly at the juxtanuclear region in close association with the Golgi marker Giantin (Fig. S2*A*). In μ1A KO cells, Giantin labeling itself appears slightly more dispersed, a possible consequence of changes in Golgi organization resulting from protein trafficking alterations previously reported for these cells (55). Nevertheless, CI-MPR showed a strongly dispersed pattern and reduced proximity to the Golgi marker (Fig. S2, *B* and *C*). Altered CI-MPR colocalization with Giantin was restored in μ1A KO cells expressing μ1A-GFP WT, but not in cells expressing μ1A(D174A)-GFP or μ1A(W408S)-GFP mutants (Fig. S2, *C*–*F*) that are defective in CI-MPR binding (Fig. 1). These results indicate that the interaction between CI-MPR and AP-1 through μ1A is crucial for the normal distribution of CI-MPR at steady state.

**Figure 2:**
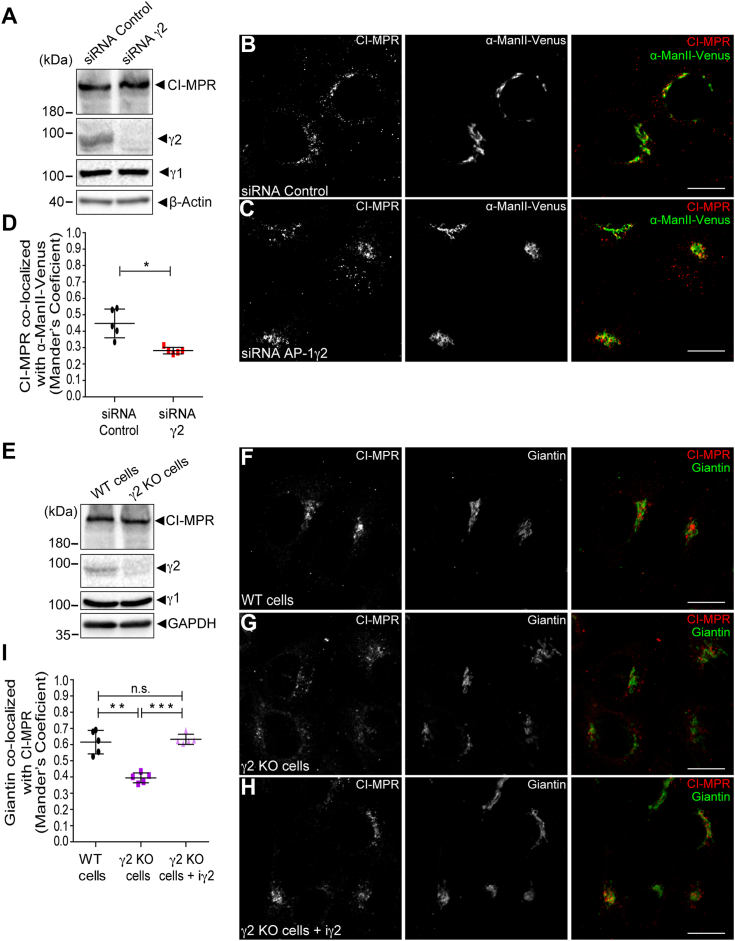
**The depletion of γ2 affects CI-MPR steady-state distribution.***A*, HeLa cells were treated twice with control siRNA or γ2 siRNA. 24 h after the second treatment, cell lysates were analyzed by Western blot. HeLa control siRNA (*B*) or γ2 KD (C) were transfected with a vector encoding α-ManII-Venus (*green channel*). After 16 h of transfection, cells were fixed and stained for CI-MPR (*red channel*). Scale bars: 10 μm. *D*, scatter plot represents the mean ± SD (n = 5) of Manders’ co-localization coefficient between the CI-MPR signal overlapping with the α-ManII-Venus signal. ∗*p* = 0.0179; (Two-tailed paired *t* test). HeLa WT cells and γ2 KO cells were analyzed by either Western blot (*E*) or immunofluorescence (*F* and *G*). HeLa WT cells (*F*), and HeLa γ2 KO cells (*G*) and γ2 KO + iγ2 cells (*H*) were fixed, permeabilized and stained for CI-MPR (*red channel*) and Giantin (*green channel*). Scale bars: 10 μm. *I*, scatter plot represents the mean ± SD (n = 5) of the Manders’ co-localization coefficient of the Giantin signal overlapping with the CI-MPR signal for each condition. ∗∗*p* = 0.0022; ∗∗∗*p* = 0.0002; n.s. = not significant (Two-tailed paired *t* test).

Subsequently, we conducted RNAi to specifically knockdown (KD) γ2 (Fig. 2*A*). Similar to what was observed in μ1A KO cells (Fig. S1*G*), the KD of γ2 does not appear to affect the overall levels of CI-MPR (Fig. 2, *A*). However, it does result in an altered steady-state distribution of CI-MPR. Compared to control cells, γ2 depletion led to a more dispersed pattern for CI-MPR and a reduced colocalization with the Golgi marker Manosidase II-Venus (56, 57) (Fig. 2, *B*–*D*).

To further confirm this phenotype, we generated a γ2 KO HeLa cell line using CRISPR/Cas9 (Fig. 2*E*) and subsequently rescued γ2 expression in these cells using a doxycycline-inducible piggyBac transposon-based expression system (58) (referred to as γ2 KO + iγ2 cells; Fig. S3). By employing this system, we successfully attained comparable levels of exogenous γ2 protein and a similar subcellular distribution pattern in γ2 knockout (KO) cells as that observed in WT cells using a doxycycline concentration of 1 ng/ml (+Dox) (Fig. S3*A*). This doxycycline concentration was consistently applied in subsequent experiments. Importantly, the ablation of γ2 did not impact the total levels or subcellular distribution of γ1 (Figs. 2*E* and S3, *B*–*E*). Similar to the observations in γ2 KD cells, depletion of γ2 in KO cells resulted in dispersed labeling of CI-MPR in cytoplasmic puncta and a notable decrease in its localization at the Golgi compared to WT cells (Fig. 2, *F*–*H*). However, this phenotype was rescued upon restoring γ2 expression in doxycycline-treated γ2 KO + iγ2 cells (Fig. 2, *H* and *I*).

The reduced juxtanuclear localization of CIMPR in γ2 depleted cells prompted us to investigate whether γ2 depletion affects the levels of CI-MPR at the cell surface. To address this, we utilized CD8-CI-MPR, a valuable reporter for studying CI-MPR trafficking, where the cytosolic tail of CD8 was replaced with the CI-MPR cytosolic tail (9, 59). Flow cytometry analysis showed that the depletion of γ2 causes an approximately 1.75-fold increase in CD8-CI-MPR levels at the cell surface (Fig. S4*A*). This suggests that impaired recycling of internalized receptors to the Golgi may cause leakage to the plasma membrane, a possibility that was tested in subsequent experiments.

Since AP-1γ1 has also been implicated in the retrograde transport of CI-MPR from endosomes to the TGN (37, 38, 39, 40, 41, 42), we tested whether AP-1γ1 and AP-1γ2 complexes co-depletion would lead to a stronger relocation of CD8-CI-MPR to the cell surface. To achieve this, we expressed CD8-CI-MPR in μ1A KO HeLa cells. Flow cytometry analysis revealed that depletion of μ1A resulted in a stronger (∼2.5-fold) increase in cell surface levels of CD8-CI-MPR (Fig. S4*B*), a phenotype that could be reversed by transfection of μ1A(WT)-GFP but not μ1A(D174A)-GFP or μ1A(W408S)-GFP mutants (Fig. S4*B*). Taken together these results suggest that both AP-1γ1 and AP-1γ2 complexes independently contribute to maintaining proper CI-MPR levels at the plasma membrane.

### CI-MPR exit from Golgi complex is not prevented by AP-1γ2 depletion

The data shown here indicates that AP-1γ2 participates in CI-MPR trafficking; therefore, we sought to investigate the possible pathways involved. To monitor the anterograde transport of CI-MPR from the TGN to early endosomes, we used the retention using selective hooks (RUSH) system. This approach entails fusing the protein of interest with a fluorescent protein and a streptavidin-binding peptide (SBP), allowing reversible entrapment of the chimera in the endoplasmic reticulum (ER) by co-expression of an ER resident protein fused to streptavidin (ER-hook). The reporter-SBP chimera can be released from the ER-hook upon the addition of biotin to the cells, enabling the tracking of its route through the secretory pathway in a synchronized manner (60).

In order to study CI-MPR trafficking using the RUSH system, we generated a stable HeLa cell line expressing streptavidin fused to the ER retention/retrieval sequence KDEL (61), and transfected these cells with a plasmid coding the transmembrane domain (TM) and the cytosolic tail (CT) of CI-MPR fused to mCherry, SBP, and a signal peptide for ER translocation at the N-terminus (RUSH-CI-MPR; Fig. S5*A*). Before biotin treatment, RUSH-CI-MPR displays a reticular pattern (0 min chase; Fig. S5*B*). Upon addition of biotin, RUSH-CI-MPR accumulates in the juxtanuclear region (50 min chase; Fig. S5*C*), and this is followed by detection of RUSH-CI-MPR in dispersed vesicles (120 min chase; Fig. S5*D*).

**Figure 3:**
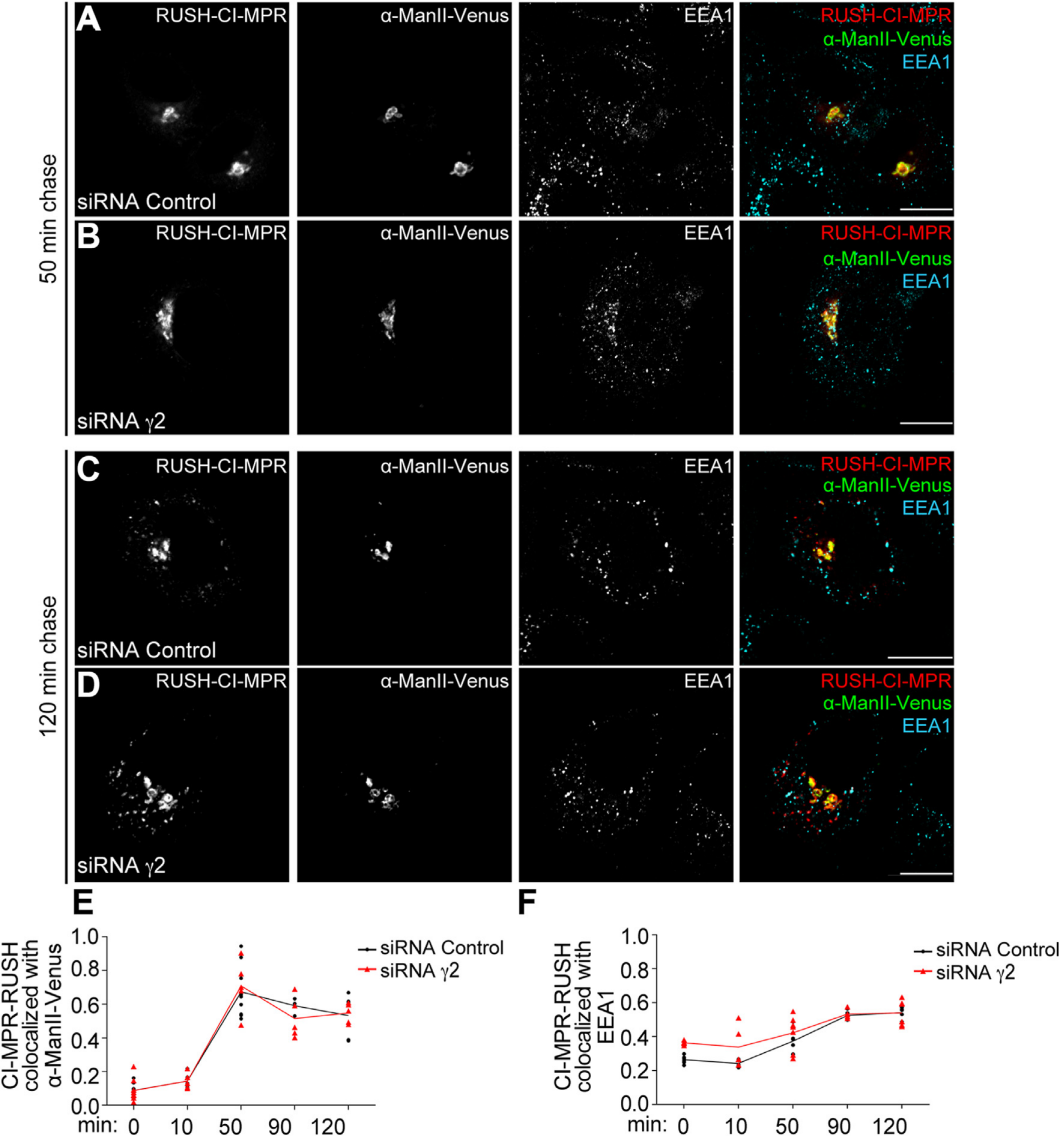
**The depletion of γ2 does not affect CI-MPR exit from the Golgi complex.** HeLa cells expressing an ER-Hook (streptavidin-KDEL) were treated with siRNA control (*A* and *C*) or γ2 siRNA (*B* and *D*) and transfected with RUSH-CI-MPR and α-ManII-Venus plasmids. After 16 h, cells were incubated with soluble biotin and fixed at different time points to follow RUSH-CI-MPR anterograde transport. *Panel A* and *B* represents cells fixed 30 min after biotin addition and *Panel C* and *D* represents the effects after 120 min of biotin addition. After fixation, cells were immunostained for EEA1 (*cyan channel*). Scale bars: 10 μm. *E* and *F*, the graphs represent the mean ± SD (n ≥ 5) of Manders’ co-localization coefficients of the RUSH-CI-MPR signal overlapping with the α-ManII-Venus signal (*E*) or with the EEA1 signal (*F*) during different time points after soluble biotin treatment.

Next, we performed γ2 KD by RNAi in cells expressing both streptavidin-KDEL and RUSH-CI-MPR. It was observed that RUSH-CI-MPR reaches the Golgi with similar efficiency in control or γ2 KD cells (Fig. 3, *A*, *B*, and *E*). After 90 min of biotin addition, the absence of γ2 did not hinder the exit of RUSH-CI-MPR from the Golgi, and it successfully reached early endosomes marked by EEA1 (Fig. 3, *C*, *D*, and *F*). These findings suggest that γ2 is not essential for the effective trafficking of CI-MPR from the Golgi to early endosomes.

### AP-1γ2 is a novel cellular factor required for CI-MPR retrograde trafficking

**Figure 4:**
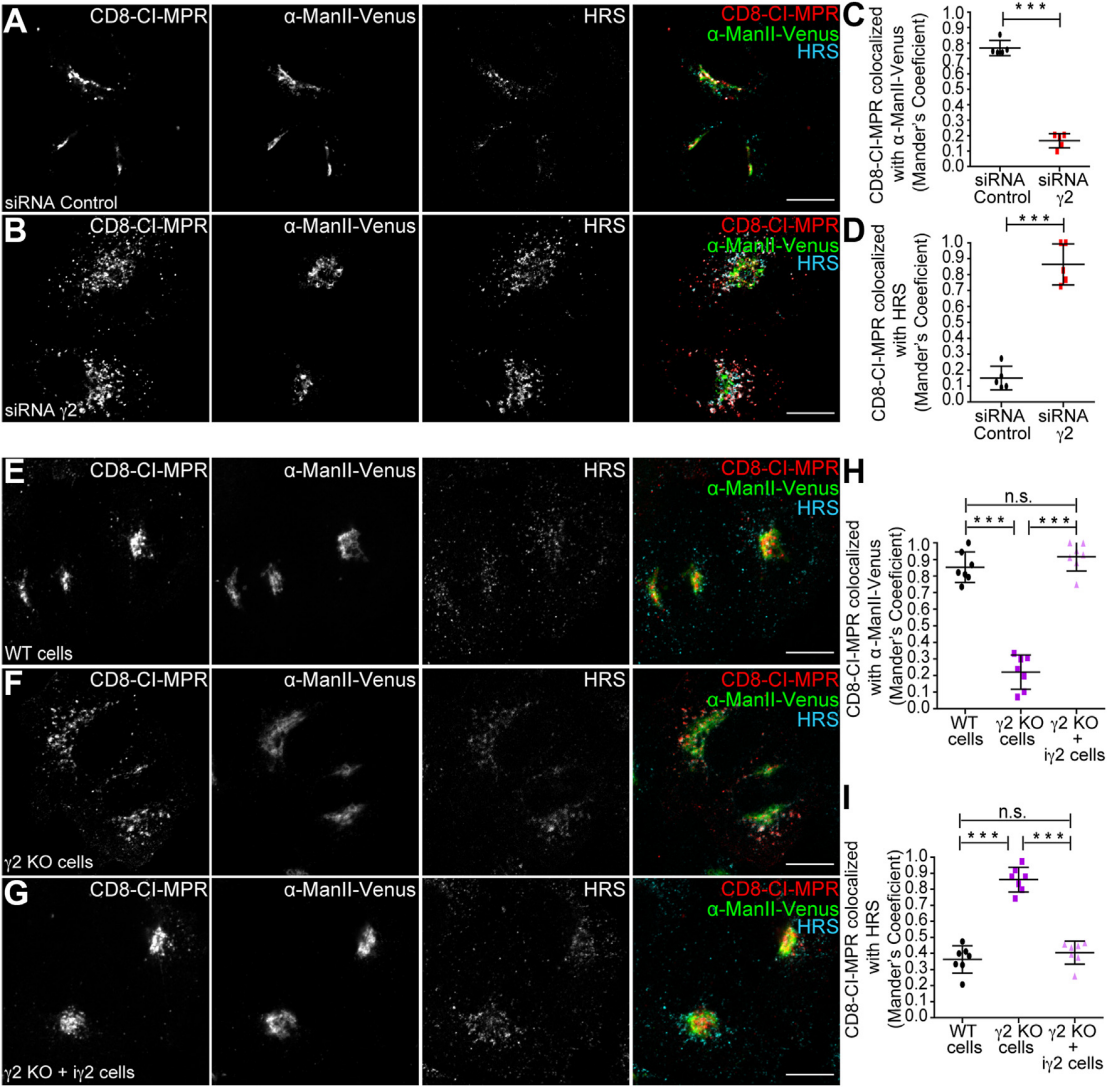
**Efficient CI-MPR retrieval from early endosomes to the Golgi complex requires γ2.** HeLa cells were treated with siRNA control (*A*) or siRNA γ2 (*B*) and then transfected with CD8-CI-MPR and α-ManII-Venus (*green channel*) plasmids. After 16 h, cells were used for anti-CD8 antibody uptake assay for 1 h (*red channel*), and then fixed and permeabilized for HRS staining (*cyan channel*). Scale bars: 10 μm. *C* and *D*, scatter plot represents the mean ± SD (n = 5) of Manders’ co-localization coefficients of the CD8-CI-MPR signal overlapping with the α-ManII-Venus signal (*C*) or the HRS signal (*D*) for each condition. ∗∗∗*p* < 0.0001 (*C*); ∗∗∗*p* = 0.0003 (*D*); (Two-tailed paired *t* test). HeLa WT cells (*E*), HeLa γ2 KO cells (*F*) or HeLa γ2 KO cells expressing exogenous γ2 in an inducible manner (+iγ2) (*G*) were transfected with CD8-CI-MPR and α-ManII-Venus (*green channel*) plasmids and used for anti-CD8 uptake (*red channel*) assays for 1 h. Cells were fixed and permeabilized for HRS staining (*cyan channel*). Scale bars: 10 μm. *H* and *I*, scatter plot represent the mean ± SD (n = 7) of Manders’ co-localization coefficients of the CD8-CIMPR signal overlapping with the α-ManII-Venus signal (*H*) or the HRS signal (*I*) for each condition. ∗∗∗*p* < 0.0001; n.s. = not significant (Two-tailed paired *t* test).

Following endocytosis, CI-MPR molecules are targeted to early endosomes and may follow a retrograde route to the TGN (7, 8, 9, 10, 11, 12). Therefore, we investigated whether AP-1γ2 is required for CI-MPR retrograde transport. To this end, we performed a previously described protocol to specifically access the retrograde transport of CI-MPR, using CD8-CI-MPR as a reporter (9, 59). Internalized CD8-CI-MPR is tracked from the cell surface by incubating living cells with an anti-CD8 antibody for defined periods, before subsequently fixing the cells for immunofluorescence analysis. After 1 h of uptake, we observed that internalized anti-CD8:CD8-CI-MPR complexes have efficiently reached the Golgi in control cells (Fig. 4, *A* and *C*). However, retrieval to the Golgi was compromised in γ2 KD cells, with retention of anti-CD8:CD8-CI-MPR complexes in early endosomes labeled by HRS (Fig. 4, *B* and *D*).

The γ2-dependent retrieval of CD8-CI-MPR from early endosomes to the Golgi was confirmed in γ2 KO and γ2 KO + inducible γ2 cells (Fig. 4, *E*–*I*). Even after a 3-h uptake period, the colocalization of CD8-CI-MPR with ManII-Venus stays consistently lower in γ2 KO cells compared to the control, while colocalization with HRS remains higher (Fig. S6). Therefore, prolonged retention of endocytosed CD8-CI-MPR in early endosomes in γ2 KO cells is likely due to a blockade in retrograde transport rather than a trafficking delay. Impairment of endosome-to-Golgi retrieval of endocytosed CD8-CI-MPR was also observed and appeared stronger in μ1A KO cells (Fig. S7). The data supports previous reports that μ1A functions in MPR retrograde transport (39, 40), and is consistent with the notion that both AP-1γ1 and AP-1γ2 complexes individually contribute to CI-MPR retrograde transport.

**Figure 5:**
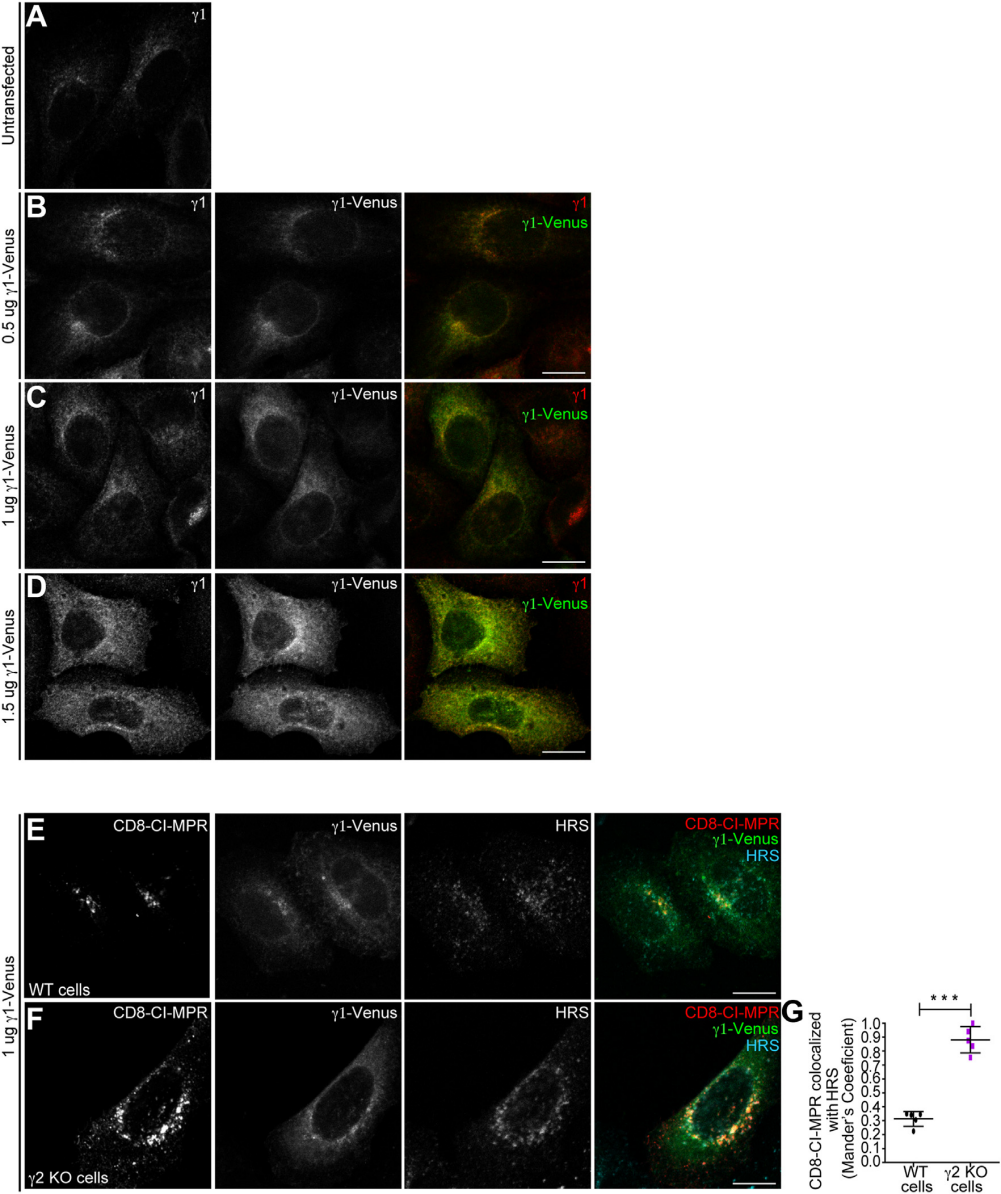
**Overexpressing exogenous γ1 in γ2 KO cells does not recover CD8-CI-MPR retrograde transport.***A*–*D*, heLa WT cells untransfected (*A*) or transfected with 0.5 μg (*B*), 1.0 μg (*C*) or 1.5 μg (*D*) of vector coding for γ1-Venus (*green channel*). After 16 h, cells were fixed and permeabilized for γ1 staining (*red channel*). Note that γ1 is miscolocalized upon transfection of 1.5 μg of vector coding for γ1-Venus. Scale bars: 10 μm. HeLa WT cells (*E*) and HeLa γ2 KO cells (*F*) were transfected with CD8-CI-MPR and 1 μg of γ1-Venus (*green channel*) plasmids. After 16 h, cells were used for anti-CD8 antibody uptake assay for 2 h (*red channel*), and then fixed and permeabilized for HRS staining (*cyan channel*). Scale bars: 10 μm. *G*, scatter plot represent the mean ± SD (n = 5) of Mander’s co-localization coefficients of the CD8-CI-MPR signal overlapping with the HRS signal for each condition. ∗∗∗*p* = 0.0003. Two-tailed paired *t* test.

Altogether, our data indicate a critical role of AP-1γ2 in the CI-MPR retrograde pathway that AP-1γ1 does not compensate for. To investigate this further, we asked if the overexpression of γ1 can suppress the retrograde trafficking defect caused by γ2 depletion. Initially, we assessed the maximum level of exogenous γ1 expression (γ1-Venus) to be employed in these assays, ensuring that it does not result in miscolocalization. At low expression levels (0.5 μg of coding vector), γ1-Venus displayed a punctate pattern that was mostly juxtanuclear, and similar to the distribution pattern of endogenous γ1 in untransfected cells (Fig. 5, *A* and *B*). At higher expression levels (1.0 and 1.5 μg of coding vector) γ1-Venus appear gradually more dispersed (Fig. 5, *C* and *D*). Hence, we utilized intermediate concentrations (1.0 μg) of the γ1-Venus coding vector for uptake experiments in γ2 KO cells. The results showed that γ1 expression does not restore the CD8-CI-MPR retrograde trafficking defect induced by γ2 depletion (Fig. 5, *E*–*G*), thereby confirming that AP-1γ2 and AP-1γ1 are not functionally redundant.

### In AP-1γ2 KO cells, endocytosed CI-MPR is sequestered in endosomes that exhibit enrichment of retromer subunits

One of the most extensively studied machineries responsible for CI-MPR retrograde transport is the retromer complex (9, 12, 62). A previous study showed that endocytosed CD8-CI-MPR transiently colocalizes with the retromer subunit Vps26 in endosomes en route to the Golgi (9). To gain insights into the interplay between the retrograde transport steps mediated by retromer and AP-1γ2, we stained endogenous retromer subunits in AP-1γ2 knockout (KO) cells during CD8-CI-MPR internalization experiments.

**Figure 6:**
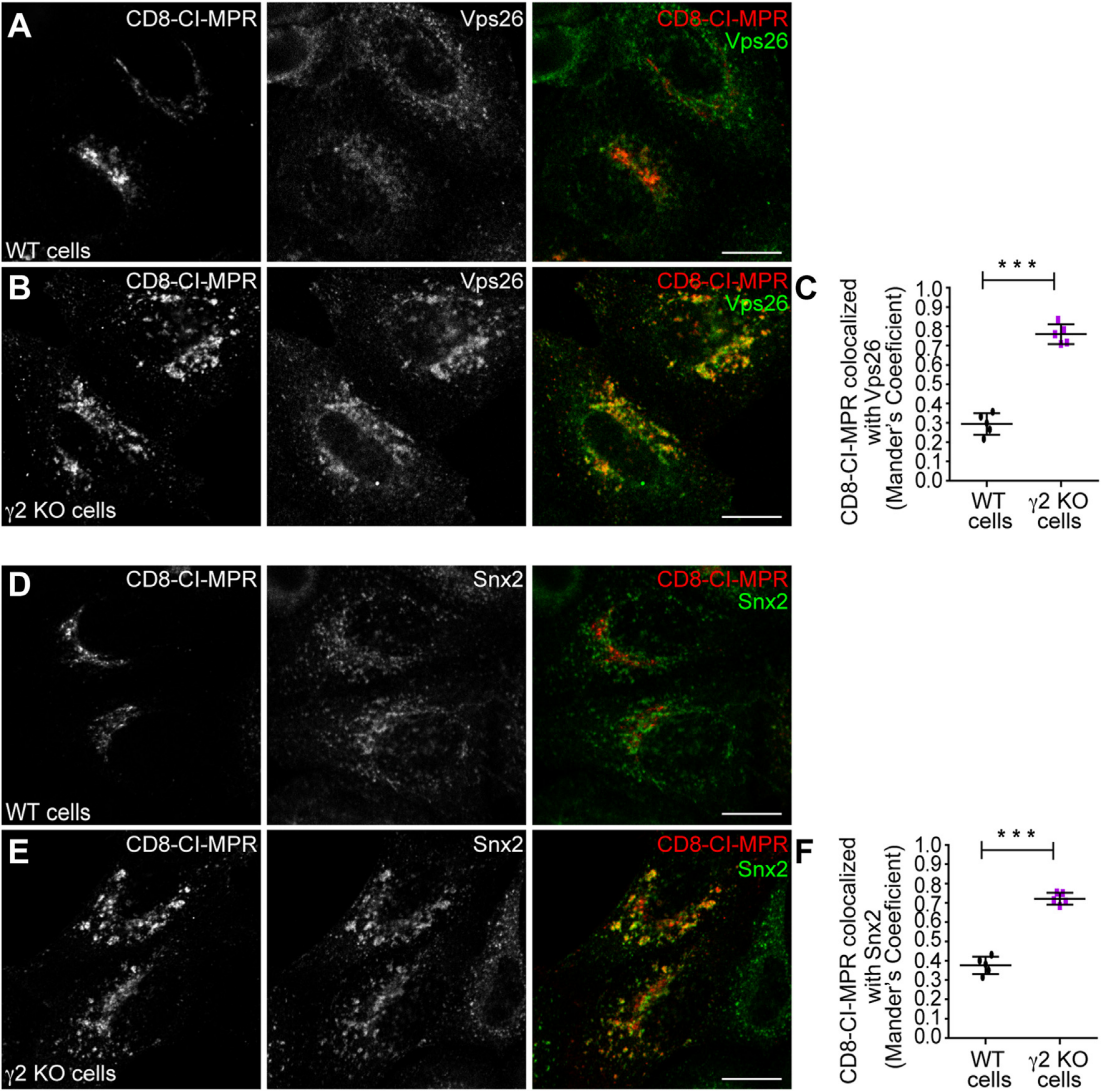
**Depletion of γ2 leads to retention of internalized CD8-CI-MPR in endosomes positive for Retromer complex subunits.** HeLa WT cells (*A* and *D*) and HeLa γ2 KO cells (*B* and *E*) were transfected with a CD8-CI-MPR plasmid. After 16 h, cells were used for an anti-CD8 antibody uptake assay for 2 h (*red channel*), and then fixed and permeabilized for Vps26 (*A* and *B*) or Snx2 (*D*–*E*) staining (*green channel*). Scale bars: 10 μm. Scatter plot represent the mean ± SD (n = 5) of Mander’s co-localization coefficients of the CD8-CI-MPR signal overlapping with the Vps26 (*C*) or Snx2 (*F*) signals for each condition. ∗∗∗*p* < 0.0001 (*C*); ∗∗∗*p* = 0.0005 (*F*) (Two-tailed paired *t* test).

In control cells, staining of Vps26 (Fig. 6, *A*–*C*) and Snx2 (Fig. 6, *D*–*F*), another retromer subunit, shows a discrete punctate pattern dispersed throughout the cytoplasm, and display relatively low colocalization with internalized CD8-CI-MPR after 2 h of uptake (Fig. 6, *C* and *F*). In γ2 KO cells, internalized CD8-CI-MPR shows a much higher colocalization with Vps26 (Fig. 6, *A*–*C*) and Snx2 (Fig. 6, *D*–*F*), which are enriched in larger vesicular structures. This suggests that CD8-CIMPR resides for an extended period in retromer-positive domains of endosomes, implying that AP-1γ2 influences the retromer dynamics.

### ATP7B retrograde transport requires AP-1γ2

**Figure 7:**
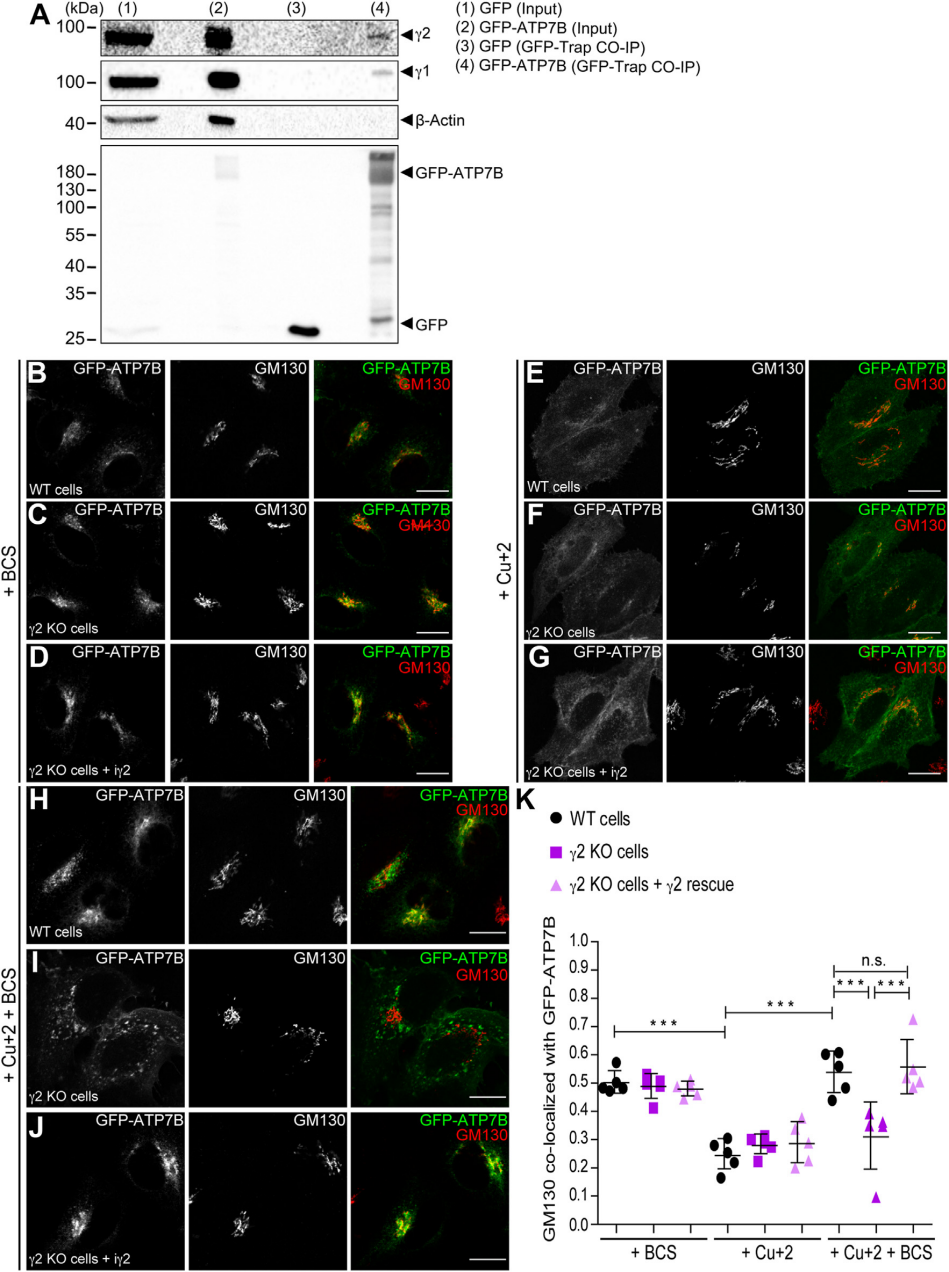
**GFP-ATP7B interacts with AP-1γ1 or AP-1γ2 and its retrieval to the TGN requires AP-1γ2.***A*, PEAK cells expressing GFP or GFP-ATP7B were treated for 2 h with 200 μM CuCl_2_ and the cleared cell lysates were subjected to GFP-Trap Co-IP assay and Western blot for GFP, γ2 and γ1. Western blot of β-actin was used as a negative control. Input represents 2% of cell lysates used in the GFP-Trap Co-IP assay. HeLa WT cells (*B*, *E*, and *H*) and HeLa γ2 KO cells (*C*, *F*, and *I*) and γ2 KO + iγ2 cells (*D*, *G*, and *J*) expressing GFP-ATP7B were treated with BCS (*B*–*D*), CuCl_2_ (*E*–*G*) and CuCl_2_ + BCS (*H*–*J*) and then fixed and permeabilized for GM130 staining (*green channel*). Scale bars: 10 μm. *K*, scatter plot represent the mean ± SD (n = 5) of Mander’s co-localization coefficients of the GM130 signal overlapping with the GFP-ATP7B signal for each condition. ∗∗*p* < 0.005 ∗∗∗*p* < 0.0005. n.s. = not significant (one-way ANOVA with Bonferroni’s corrections).

To further explore the involvement of AP-1γ2 in retrograde trafficking from endosomes to the Golgi complex, we investigated the trafficking of the copper transporter ATP7B, known to rely on AP-1 (37, 44, 45, 47). Previous studies demonstrated the interaction of ATP7B with AP-1 (44, 45, 47). Co-immunoprecipitation assays using GFP-Trap showed the interaction of both γ1 and γ2 with GFP-ATP7B (Fig. 7*A*), suggesting that either AP-1 variant can bind ATP7B.

The interaction between ATP7B and AP-1γ2 prompted us to investigate the role of this AP-1 variant in ATP7B intracellular trafficking. To track ATP7B trafficking, we transfected GFP-ATP7B into HeLa WT, γ2 KO, or γ2 KO + iγ2 cells (Fig. 7, *B*–*K*). Under copper-limiting conditions (+BCS), GFP-ATP7B predominantly localized to the juxta nuclear region, colocalizing with the Golgi marker GM130 in all three cell lines (Fig. 7, *B*–*D*, and *K*). In contrast, high levels of Cu+ (200 μM) resulted in the redistribution of GFP-ATP7B from the Golgi complex to the cell surface, reducing its colocalization with GM130 (Fig. 7, *E*–*G*, and *K*).

Consistent with the findings from RUSH-CI-MPR (Fig. 3), the depletion of γ2 did not affect the egress of GFP-ATP7B from the Golgi complex (Fig. 7*F*), suggesting that AP-1γ2 may not be involved in this anterograde pathway. Importantly, after copper washout, GFP-ATP7B relocated from the cell surface back to the Golgi complex (Fig. 7, *H* and *K*), a process impaired in γ2 KO cells (Fig. 7, *I* and *K*), but rescued by the induced expression of γ2 (Fig. 7, *J* and *K*). Overall, these results support the requirement of AP-1γ2 for ATP7B retrograde trafficking.

## Discussion

Our study provides compelling evidence that the γ2-adaptin is an integral component of a functional AP-1 complex variant, serving as novel sorting machinery that regulates the trafficking of both CI-MPR and ATP7B, and that is not functionally compensated by γ1. Specifically, we establish the crucial role of AP-1γ2 in mediating the retrograde transport of transmembrane receptors from the endosomal system back to the TGN. While previous studies have implicated AP-1 in the bidirectional transport of CI-MPR between the TGN and endosomes (34, 35, 36, 37, 38, 39, 40, 41, 42), these investigations focused on perturbing the μ1 and/or γ1 subunits, without exploring the involvement of the γ2 isoform. Our findings shed new light on the functional significance of a poorly characterized variant of AP-1 in these critical transport processes.

Our initial experiments demonstrated the interaction of CI-MPR with both AP-1γ1 and AP-1γ2 complexes (Fig. 1). Utilizing yeast two-hybrid and Co-IP assays, we confirmed that CI-MPR directly binds to the μ1 subunit. Additionally, we established that the conserved YXXΦ motif in the cytosolic tail of CI-MPR (_2353_YSKV_2356_) is responsible for its interaction with μ1A, specifically through the tyrosine binding pocket. While the binding of μ1A-(WT)-GFP to γ1 or γ2 subunits does not rely on the integrity of the tyrosine binding pocket, our experiments with mutants in this region of μ1A revealed a decreased ability to capture (Fig. 1) and transport (Fig. S2) CI-MPR, indicating that the interaction with μ1A is crucial for stabilizing the CI-MPR complex with AP-1.

To further support the involvement of γ2-adaptin in an AP-1 complex variant, we investigated the stability and subcellular distribution of γ2 in μ1A KO cells. Consistent with our previous findings (51, 52), we observed a significant reduction in the levels of γ2 and the loss of its punctate distribution pattern in μ1A KO cells (Fig. S1). Notably, the levels of γ2 and its subcellular distribution were restored when exogenous μ1A-GFP WT or μ1A-GFP tyrosine binding pocket mutants were expressed in μ1A KO cells. Similar results were also observed for γ1 (Fig. S1), indicating that the stability and localization of both γ1 and γ2 are dependent on the presence of μ1A.

Before investigating the specific role of AP-1γ2 in CI-MPR distribution, we used μ1A KO cells to examine the effects of AP-1γ1 and AP-1γ2 co-depletion. In line with the established role of μ1A in the retrieval of CI-MPR from endosomes to the Golgi (39), CI-MPR localization shifted from a perinuclear to a more dispersed punctate pattern in μ1A KO cells (Fig. S2). Moreover, μ1A KO cells misdirected a CD8-CI-MPR chimera to the plasma membrane (Fig. S4). These effects are likely to be direct consequences of AP-1 function in CI-MPR trafficking itself, since expression of μ1A mutants able to form complexes with the γ-subunits, but that does not bind CI-MPR efficiently, are unable to rescue the defective sorting phenotypes, in contrast to exogenous μ1A WT expression (Figs. S2 and S4).

The specific depletion of AP-1γ2 resulted in a dispersed intracellular distribution of endogenous CI-MPR compared to control cells (Fig. 2). This phenotype was confirmed by γ2 RNAi and KO/rescue strategies and is consistent with previous studies linking dispersed CI-MPR localization to impaired endosome-to-TGN sorting (63, 64, 65). Further supporting this notion, the retrieval of CD8-CI-MPR from endosomal compartments to the Golgi complex was compromised in AP-1γ2-depleted cells, as the internalized reporter construct failed to reach the Golgi and instead accumulated in early endosomes that contain HRS (Figs. 4 and 5) and retromer subunits (Fig. 6). These findings suggest that AP-1γ2 may play a broader role in the retrograde pathway. This is further supported by the observation that GFP-ATP7B, a cargo known to require AP-1 for retrograde trafficking, failed to return from the cell surface to the Golgi complex in γ2 knockout cells and instead became trapped in endosomes (Fig. 7).

Significantly, the missorting phenotypes of CI-MPR observed in μ1A KO cells (Figs. S2 and S6), where both γ1 and γ2 are depleted, were stronger than those observed when γ2 alone is lacking (Figs. 2 and 4). However, the observed partial impairment in retrograde trafficking in γ2 KO cells does not solely arise from a reduction in the overall concentration of either AP-1 variant. This conclusion is supported by the fact that augmenting the intracellular levels of γ1 in these cells through exogenous expression did not restore the retrograde trafficking defect caused by γ2 depletion (Fig. 5).

Previous data suggest that AP-1γ1 mediates the anterograde transport of MPRs in cooperation with GGAs (Golgi-localized, γ-adaptin ear-containing, Arf-binding) adaptors (37, 66, 67). In addition, the interaction of ATP7B with AP-1γ1 has been shown to affect ATP7B localization at the TGN (44, 45, 47). Interestingly, our findings indicate that the depletion of AP-1γ2 does not impair the efficient exit of CI-MPR and ATP7B from the Golgi complex, as observed in synchronized anterograde trafficking assays (Figs. 5 and 7). Furthermore, while AP-1γ1 is crucial for sorting cargo proteins to the basolateral and apical membranes in polarized cells, AP-1γ2 appears to have distinct functions (68). In conclusion, our study contributes to a growing body of evidence showing that AP-1γ1 and AP-1γ2 are not merely redundant AP-1 complexes (69).

Why does AP-1γ2 depletion compromise CI-MPR and ATP7B retrograde transport? Clathrin and clathrin-related adaptor proteins have previously been implicated in endosome-to-TGN trafficking. Monomeric adaptor proteins, such as epsinR and GGAs, have been shown to facilitate the retrieval of cargo proteins from endosomes (46, 70, 71, 72). Moreover, the μ1 and γ1 subunits of AP-1 have been implicated in the retrograde pathway of CI-MPR (37, 41, 42) and ATP7B (37, 47, 73) from endosomes to the TGN. Therefore, the depletion of AP-1γ2 may disrupt the recruitment of clathrin and its adaptors to specific subdomains of endosomes involved in retrograde transport, leading to impaired CI-MPR and ATP7B transport from these organelles.

We propose that both AP-1γ1 and AP-1γ2 variants play independent roles in facilitating the proper transport of CI-MPR and ATP7B from the endosomal system back to the Golgi complex. Cargo proteins employ multiple sorting machinery elements to achieve accurate membrane localization within the endo-lysosomal system. However, how these different sorting machinery components are organized, and their functions coordinated to perform the endosome-to-TGN transport remains a subject of debate (63, 64, 65, 74). The retromer complex is the most extensively studied sorting machinery involved in retrograde endosome-to-TGN trafficking, acting as a transport carrier coat and as a central hub that recruits diverse molecules to early endosomes (75, 76, 77).

A previous study suggested that clathrin and retromer act in two successive steps of the same retrograde pathway from endosomes to the TGN (78). According to this model, the recruitment of clathrin onto maturing early-endosomal microdomains is necessary to initiate membrane-curvature changes, ultimately leading to tube formation. This process is essential for the efficient retromer-mediated transport to the TGN. Our findings align with this model and indicate that the function of AP-1γ2 influences retromer dynamics. In cells lacking AP-1γ2, the internalized CD8-CI-MPR fails to reach the Golgi; instead, it accumulates in enlarged endosomes enriched for VPS26 and SNX2 (Fig. 6), suggesting that AP-1γ2 and retromer are involved in sequential trafficking step within the same transport pathway. In this context, a systematic investigation into whether and how AP-1γ2 influences the localization of Rab proteins involved in specific trafficking pathways between endosomes and the TGN (79, 80) may offer invaluable additional insights into AP-1γ2 function in these processes.

The physical proximity of the retromer complex to clathrin-coated structures at endosomes has been reported, and retromer complex subunits have been found in CCV preparations (78, 81, 82, 83). On the other hand, the retromer subunits and clathrin are present in distinct transport intermediates, and there is no evidence of direct interaction between clathrin/adaptors and retromer subunits (84, 85). In this scenario, ESCPE-1, a membrane-tubulating complex formed by heterodimers of SNX1 or SNX2 and SNX5 or SNX6, was also shown to facilitate endosome to TGN transport of CI-MPR independently of retromer (86, 87). Intriguingly, µ1 (Fig. 1) and the SNX5/SNX6 subunit of ESCPE-1 (88) bind to partially overlapping regions of the CI-MPR tail. Therefore, unraveling whether these molecules and complexes function independently or collaboratively and whether they act concurrently or sequentially remains challenging in the field.

The accumulation of CI-MPR and ATP7B in endosomes upon AP-1γ2 depletion could also be attributed to its proposed role in endosomal maturation through interactions with subunits of the Endosomal Sorting Complex Required for Transport (ESCRT) machinery (50). Specifically, AP-1γ2, but not AP-1γ1, is crucial for the efficient targeting of the EGF:EGFr complex to the canonical multivesicular bodies (MVBs) pathway (49, 50, 51). In addition, AP-1γ2 is utilized by HIV-1 Nef to retain CD4 and MHC-1 in endosomes and promote their lysosomal degradation (51, 52), which is known to involve targeting to MVBs (89, 90). These observations suggest that AP-1γ2 plays a unique role on endo-lysosomal function and trafficking processes.

## Experimental procedures

### Plasmids

For yeast two-hybrid assays, the plasmids containing the full-length sequences of mouse μ1A and its D174A and W408S mutants, mouse μ2, rat μ3A, human μ4 and the C-terminal domain of human μ1B (residues 137–423), subcloned in the pACT2 vector in fusion with the Gal4-activation domain (AD) (Clontech) have been described previously (54, 91). The CI-MPR cytosolic tail (last 164 amino acids) was subcloned from the pB42AD vector (12, 92) into the pBridge vector (Clontech), and the pBridge-CI-MPR_CT(Y26A) was generated in this study by site-directed mutagenesis (QuickChange - Agilent) and was verified by sequencing. The cytosolic tails of TGN38 and LAMP-2a were subcloned in fusion with the Gal4-binding domain (BD) in the vectors pGBT9 and pBridge, respectively (29, 31). The plasmid used to express μ1A-GFP was previously described (91) and encodes a GSGSGGSGSGRDPPVAT amino acid linker between the μ1A and GFP sequences. The plasmid used to express GFP-ATP7B was previously described (45). All of the above plasmids were kindly donated by Juan Bonifacino (NICHD, NIH). The plasmid to express γ1-Venus was obtained by subcloning the human γ1 coding sequence into the *XhoI/Bam*HI restriction sites of pVenus-N1 vector (Clontech), resulting on a RDPPVAT amino acid linker between the γ1 and Venus sequences. The plasmid for α-ManII-Venus was kindly donated by George Patterson and was previously described (56). The coding sequence for IL-2s.s.-SBP-mCherry was amplified by PCR from the mCherry-APP-GFP RUSH bicistronic vector (93) and inserted into the *NheI*/*EcoRI* sites of the pCI-neo vector (Promega; here after named pCI-neo_RUSH). Then, the coding sequence for the transmembrane and cytosolic tail of CI-MPR was amplified by PCR from the GFP-CIMPR construct, kindly donated by Juan Bonifacino, and cloned in frame with IL-2s.s.-SBP-mCherry at *EcoRI/SalI* of the pCI-neo_RUSH, resulting in the RUSH-CI-MPR construct used in the experiments. To allow inducible expression of γ2 in HeLa cells using a piggyBac transposon-based mammalian cell expression system (58), the human coding sequence for γ2 was amplified by PCR from the pGADT7_γ2 (51) and inserted into the *Nhe*I*/Hind*III sites of the PB-TSW vector, resulting in the PB-Tγ2 construct. The original PB-TSW, a generous gift from Dr Stephen Graham (University of Cambridge), is derived from PB-T-PAF (58) after the insertion of a woodchuck hepatitis virus (WHV) posttranscriptional regulatory element (WPRE) at the 3′ end. The pF5A_PBase plasmid encoding the piggybac transposase (PBase) was also generated by Dr Stephen Graham by subcloning the PBase sequence from pPBase (58), into the pF5A vector from Promega. The PB-RN plasmid carrying the reverse tetracycline transactivator (rtTA) inducer and the neomycin resistance gene was previously described (58). The construct coding the cytoplasmic tail of CIMPR fused to the extracellular and transmembrane domains of CD8 (CD8-CI-MPR) was a kind gift from Matthew Seaman, University of Cambridge (9).

### Yeast two-hybrid assay

Yeast two-hybrid assays were performed using the yeast AH109 reporter strain as described previously (51). Yeast containing both plasmids with Gal4 Activation Domain (AD) or Binding Domain (BD) was selected in a medium without tryptophan and leucine. Protein interactions were determined by yeast growth in a selective medium lacking leucine, tryptophan, and histidine.

### Immunoprecipitation assay of endogenous CI-MPR

Immunoprecipitation assays were performed as previously described (94). Briefly, HeLa cells were lysed with PBS-T (137 mM NaCl, 2.7 mM KCl, 10 mM Na_2_HPO_4_, 1.76 mM KH_2_PO_4_, and 0.1% [vol/vol] Triton X-100 [Sigma-Aldrich], pH 7.4; supplemented with a protease inhibitor cocktail) on ice for 20 min and then clarified by centrifugation at 14,000*g* for 20 min at 4 °C. Next, cell lysate was precleared with protein G-Sepharose (17–0618–01; GE Healthcare) beads for 2 h at 4 °C under orbital agitation. Precleared lysates were recovered and incubated with mouse CD4 antibody (MHCD0400–4; Invitrogen) or mouse CI-MPR antibody (Ab2733; Abcam) for 2 h followed by a further 2 h incubation with protein G-Sepharose beads, under orbital agitation at 4 °C. The samples were then washed five times in PBS-T and the immunoprecipitated complexes were eluted with sample buffer [SDS 4%, Tris-HCl 160 mM (pH 6.8), glycerol 20%, DTT 100 mM and 0.005% Bromphenol Blue] and used for SDS-PAGE and Western blot analysis.

### GFP-Trap Co-IP assay

GFP-Trap agarose (ChromoTek) assays were performed following the manufacturer's recommendations. Briefly, 85% confluent PEAK cells placed in a 100 mm plate were transfected with vectors to express GFP, AP-1μ1A(WT)-GFP, AP-1μ1A(D174A)-GFP or AP-1μ1A(W408S)-GFP by using 30 μl of 25-kDa linear PEI (1-mg/ml stock solution). After 16 h, cells were resuspended in lysis buffer [10 mM Tris/HCl pH 7.5, 150 mM NaCl, 0.5 mM EDTA, 0.5% Nonidet P40, supplemented with a protease inhibitor cocktail (P8340; Sigma-Aldrich)] and centrifuged at 14,000*g* for 20 min at 4 °C. Protein levels in the clarified cell lysates were quantified using the Bio-Rad’s Bradford protein assay (Biorad) to equalize total protein levels. Equal amounts of proteins were incubated with GFP-Trap beads for 2 h at 4 °C while rotating. Next, beads with bound proteins were washed 5 times with washing buffer [10 mM Tris/HCl (pH 7.5), 150 mM NaCl, 0.5 mM EDTA], eluted with sample buffer [160 mM Tris-HCl (pH 6.8), 4% SDS, 20% glycerol, 100 mM DTT and Bromphenol Blue 0.005%], boiled, and analyzed by SDS-PAGE and Western blot. For GFP-ATP7B Co-IP assay, a protocol was adapted from the previous work of Lalioti *et al.* (44). Briefly, 85% confluent PEAK cells placed in two 100 mm plates were transfected with vectors to express GFP or GFP-ATP7B by using 30 μl of 25-kDa linear PEI (1 mg/ml stock solution). After 20 h, cells were incubated with 200 μM CuCl_2_ for 2 h at 37 °C. After this period, cells were resuspended with lysis/wash buffer [20 mM HEPES pH 7.5, 250 mM Sucrose, 0,1% Nonidet P40, 130 mM NaCl, 1 mM EDTA, 1 mM Sodium Orthovanadate, 50 mM Sodium Fluoride, 100 mM Sodium pyrophosphate, supplemented with a protease inhibitor cocktail (P8340; Sigma-Aldrich)] and centrifuged at 14,000*g* for 20 min at 4 °C. Protein levels in the clarified cell lysate were quantified using Bio-Rad’s Bradford protein assay (Biorad) to equalize total protein levels. Equal amounts of proteins were incubated with GFP-Trap beads overnight at 4 °C while rotating. Next, beads with bound proteins were washed 5 times with lysis buffer/wash buffer and then eluted with the sample buffer described above, boiled, and analyzed by SDS-PAGE and Western blot.

### Cell culture, transfections, and siRNA

PEAK cells (HEK-293T cells transfected with the large T antigen of SV-40) were grown as previously described (95). HeLa AP-1μ1A CRISPR/Cas9 KO and HeLa WT cells were a gift from Margaret S. Robinson (University of Cambridge) and have been previously described (55). Cells were expanded, frozen as aliquots and stored in vapor phase liquid nitrogen. The aliquots were used up to the 15th passage and discarded. PCR tests for *mycoplasma* were negative. For RNA interference assays, HeLa cells were subjected to two rounds of siRNA transfection with a 48 h interval, using the Oligofectamine reagent (Thermo Scientific). The siRNAs were purchased from Dharmacon as nucleotide duplexes with 3′dTdT overhangs, designed to target human AP-1γ2 (5′-AAACCCUGCUUUGCUGUUAA-3′) (51). In the control experiments, the MISSION siRNA Universal Negative Control (SIC001, Sigma-Aldrich) was used. Cells were harvested for the assays 4 days after the first siRNA transfection. Alternatively, at day 4, cells were transfected with plasmids DNA using Lipofectamine 2000 (Thermo Scientific) and analyzed on day 5.

### Generation of γ2 KO cells by CRISPR/Cas9 and γ2 KO cells rescued for γ2 expression

The identification of a gRNA target in exon 1 (5′-CCTCATCGAAGAGATTCGCG-3′) of the AP1G2 gene was performed by using the Zhang online CRISPR design tool (http://crispr.mit.edu/) (96). The gRNA was ordered as a pair of complementary oligonucleotides (Sigma-Aldrich) with the sequences 5′-CACCGN20 to 3′ and 5′-AAACN20C-3′, annealed and cloned into the *Bbs*I site of the pX330 vector (Addgene), which expresses both Cas9 and gRNA. HeLa cells were transfected with pX330 and pIRESpuro (Clontech) in a ratio of 3:1 using the TransIT-HeLaMONSTER transfection kit (MIR 2900; Mirus Bio). After 2 days, cells were treated with Puromycin for 3 days and single-cell clones were isolated by serial dilution and tested for γ2 KO by Western blot and immunofluorescence assays. To generate γ2 KO cells rescued for γ2 expression (termed γ2 KO + iγ2 cells), we transfected the PB-Tγ2, PB-RN, and pF5A_PBase in γ2 KO cells (ratio 5:1:1) by using Lipofectamine 2000 (Thermo Scientific). After 16 h post-transfection, cells were treated with Puromycin (P8833; Sigma-Aldrich) and Geneticin (11,811–031; Gibco) for 1 week and different concentrations of Doxycycline (D9891; Sigma-Aldrich) were tested to induce γ2 expression.

### Antibodies

The mouse anti-CI-MPR antibody (Ab2733; Abcam) and rabbit anti-CI-MPR antibody (ab124767; Abcam) were used for immunofluorescence and Western blot, respectively. The antibodies for Giantin (924,302; Convance), HRS (ab155539; Abcam), GM130 (610,822; BD Biosciences) and EEA1 (610,456; BD Biosciences) were used for immunofluorescence. The antibodies for γ1 (610,386; BD Biosciences), γ2 (HPA004106; Sigma-Aldrich), μ1A (AB111135; Abcam), GAPDH (G9545; Sigma-Aldrich) and β-actin (sc-47778; Santa Cruz) were used for Western blot. The same antibodies for γ1 and γ2 were also used for immunofluorescence. The rabbit anti-Vps26 and anti-Snx2 anti-serum were kind gifts from Juan Bonifacino, and previously described (97, 98). The rabbit anti-GFP antibody used for western-blot was a gift from R. Hegde (MRC). The mouse monoclonal anti-CD8 antibody used for flow cytometry and antibody uptake assays was a kind gift from Matthew Seaman (MRC) (9). Horseradish-peroxidase-conjugated donkey anti-mouse immunoglobulin G (IgG) and donkey anti-rabbit IgG were obtained from GE Healthcare. Secondary antibodies conjugated to Alexa fluorophores were purchased from Thermo Scientific.

### Anti-CD8 antibody uptake assay

The anti-CD8 antibody uptake assay was performed as previously described (9, 59, 94). Briefly, HeLa cells expressing CD8-CI-MPR were washed 3 times with PBS and incubated with Opti-MEM for 30 min at 37 °C. Then, cells were incubated for 1h to 3h (see Figure legends) continuously with the anti-CD8 antibody (1 μg of anti-CD8 antibody in DMEM), depending on the experiment, as described in the Figure legends. Cells were washed 3 times with PBS, fixed with PFA 4% in PBS, and processed for immunofluorescence as described below.

### Retention using selective hooks (RUSH) assay and immunofluorescence microscopy

HeLa cells transfected with vectors coding for ER-hook and RUSH-CI-MPR were processed for single cell sorting by the Becton Dickinson FACS Aria Fusion (Center for Cell-Based Therapy/Hemocentro de Ribeirão Preto) selecting cells positive for the mCherry signal. mCherry-positive cells were grown under puromycin and G418 treatment and individual clones were used in RUSH assays. Cells were treated with 40 μM of soluble Biotin (pulse) and incubated for different time points (chase) prior to fixation. Immunofluorescence was performed as previously described with no modifications (99). Cells were imaged on a Zeiss confocal laser-scanning microscope (LSM) 780 (Zeiss). Post-acquisition image processing and co-localization analysis were as previously described (51).

### GFP-ATP7B trafficking assay

The GFP-ATP7B trafficking assay was adapted from previous work from Jain and colleagues (45). Briefly, HeLa WT cells, γ2 KO cells and γ2 KO + iγ2 cells were grown on coverslips and transfected with pGFP-ATP7B using Lipofectamine 2000 (Thermo Scientific). After 16 h post transfection, cells in DMEM media were treated as follow: (1) cells treated with Cu+-chelator bathocuproinedisulfonic acid (BCS) only - cells were incubated with 50 μg/ml cycloheximide and 200 μM BCS for 4 h at 37 °C (medium was replaced every 2 h); (2) cells treated with CuCl_2_ only - cells were treated with 50 μg/ml cycloheximide and 200 μM CuCl_2_ for 2 h; (3) cells treated with CuCl_2_ and BCS (copper washout) - cells were treated first with 200 μM CuCl_2_ for 2 h, and then with 200 μM BCS for further 4 h, always in the presence of 50 μg/ml cycloheximide (medium was replaced every 2 h).

### SDS-PAGE and Western blot analysis

Total cell lysates were prepared and equalized for total protein concentration, as previously described (51). Briefly, protein homogenates were mixed with sample buffer [160 mM Tris-HCl (pH 6.8), 4% SDS, 20% glycerol, 100 mM DTT, and 0.005% Bromphenol Blue], boiled, and subjected to SDS-PAGE and electrotransferred to a nitrocellulose membrane (Millipore, Bedford, MA, USA). The membranes were blocked with PBS-T (PBS, 05% Tween 20) and 5% nonfat dry milk for 1 h at room temperature, followed by individual incubation with primary antibodies in PBS + 1% BSA overnight at 4 °C under gentle agitation. After that, the membranes were washed 5 times with PBS-T and incubated with secondary antibodies conjugated to horseradish peroxidase (HRP) (GE Healthcare) in PBS-T (PBS, 05% Tween 20) and 5% nonfat dry milk for 1 h at room temperature. The membranes were washed five times with PBS-T and proteins were detected using enhanced chemiluminescence solutions [solution 1: 1 M Tris–HCl (pH 8.5), 250 mM luminol, 90 mM p-coumaric acid; and solution 2: 30% H_2_O_2_, 1 M Tris-HCl (pH 8.5)] and visualized with the ChemiDoc imaging system (GE Life Science).

### Immunofluorescence and confocal assays

Immunofluorescence was performed, as previously described (51). Briefly, cells were grown overnight on glass coverslips, then fixed with 4% (wt/vol) paraformaldehyde (PFA) in PBS for 15 min at room temperature. Fixed cells were permeabilized/blocked with blocking solution [0.01% (wt/vol) Saponin (S7900; Sigma-Aldrich), 0.2% [wt/vol] pork skin gelatin (G2500; Sigma-Aldrich) in PBS] for 15 min at 37 °C. Next, cells were incubated with primary and, subsequently, with secondary antibodies for 1 h at 37 °C in blocking solution. Coverslips were mounted on slides with Fluoromount-G Mounting Medium (17,984–25; Electron Microscopy Sciences), and cells were imaged on a Zeiss confocal laser scanning microscope (LSM) 780 (Zeiss, Jena, Germany). Post-acquisition image processing was performed with ImageJ 1.36 (100). Co-localization analyses were performed with sets of images of the same cells (Z-stack) for each marker. Quantification was performed with ImageJ and the plug-in Co-localization Threshold to determine the Mander’s coefficients (tM) for each channel.

### Flow cytometry analysis

To assess the levels of surface CD8-CI-MPR, unfixed cells were incubated for 30 min at 4 °C with mouse anti-CD8 antibody (9, 59) followed by incubation for 30 min at 4 °C with F(ab')2-Goat anti-Mouse IgG (H + L) Cross-Adsorbed Secondary Antibody Alexa Fluor 647 (Cat. A21237; Thermo Scientific) in PBS supplemented with 1% BSA, and then fixed with PFA 1% in PBS + 1% BSA and subjected to flow cytometry analysis. Data were acquired using the levels of Alexa 647 fluorescence in cells from a BD LSRFortessa flow cytometer (BD Biosciences) and FACSymphony A5 Cell Analyzer (BD Biosciences) at the Ribeirão Preto Center for Cell-Based Therapy/Hemotherapy. The FloJo software (Tree Star) was used for data analyses.

### Statistical analysis

All statistical data are demonstrated as mean ± SD and the n samples are indicated in the figure legend for each analysis. Data were plotted and analyzed using GraphPad Prism 5.0 software.

## Data availability

All data are contained within the article itself and/or its supporting information, which is accessible online.

## Supporting information

This article contains supporting information.

## Conflict of interest

The authors declare that they have no known competing financial interests or personal relationships that could have appeared to influence the work reported in this paper.
