# Supplementary material for: AP-1γ2 is an adaptor protein 1 variant required for endosome-to-Golgi trafficking of the mannose-6-P receptor (CI-MPR) and ATP7B copper transporter
Source: J Biol Chem. 2024 Feb 1;300(3):105700. doi: 10.1016/j.jbc.2024.105700 (PMC10909764; doi:10.1016/j.jbc.2024.105700)
Supplement: Supplemental Figures [file mmc1.pdf]

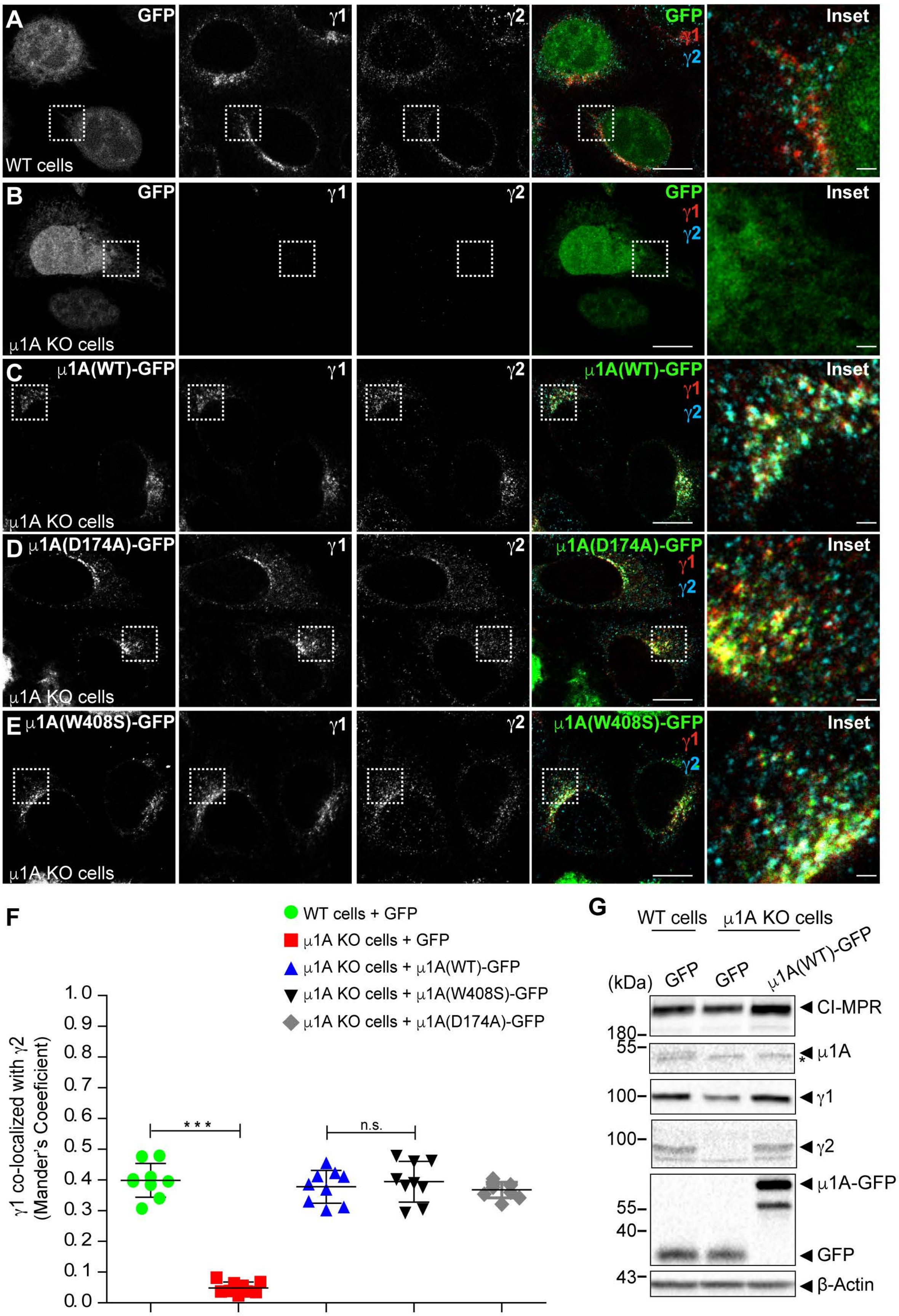
A
GFP
γ1
γ2
GFP
γ1
γ2
Inset
WT cells
B
GFP
γ1
γ2
GFP
γ1
γ2
Inset
μ1A KO cells
C
μ1A(WT)-GFP
γ1
γ2
μ1A(WT)-GFP
γ1
γ2
Inset
μ1A KO cells
D
μ1A(D174A)-GFP
γ1
γ2
μ1A(D174A)-GFP
γ1
γ2
Inset
μ1A KO cells
E
μ1A(W408S)-GFP
γ1
γ2
μ1A(W408S)-GFP
γ1
γ2
Inset
μ1A KO cells
F
WT cells + GFP
μ1A KO cells + GFP
μ1A KO cells + μ1A(WT)-GFP
μ1A KO cells + μ1A(W408S)-GFP
μ1A KO cells + μ1A(D174A)-GFP
γ1 co-localized with γ2
(Mander's Coeeficient)
1.0
0.9
0.8
0.7
0.6
0.5
0.4
0.3
0.2
0.1
0.0
***
n.s.
G
WT cells
μ1A KO cells
(kDa)
GFP
GFP
μ1A(WT)-GFP
CI-MPR
180
55
μ1A
*
100
γ1
100
γ2
μ1A-GFP
55
40
GFP
43
β-Actin

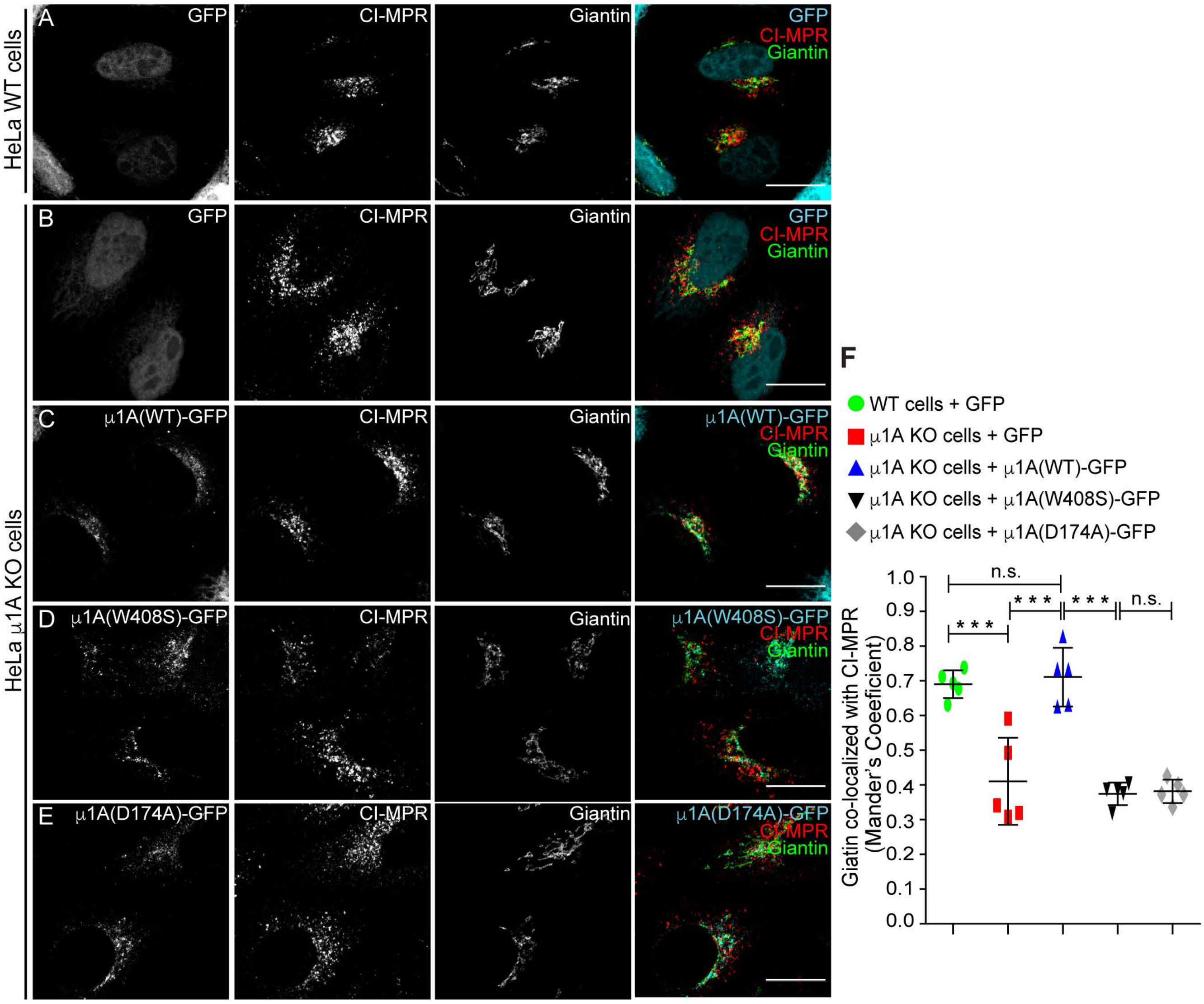

HeLa WT cells
HeLa μ1A KO cells
A
GFP
CI-MPR
Giantin
GFP
CI-MPR
Giantin
B
GFP
CI-MPR
Giantin
GFP
CI-MPR
Giantin
C
μ1A(WT)-GFP
CI-MPR
Giantin
μ1A(WT)-GFP
CI-MPR
Giantin
D
μ1A(W408S)-GFP
CI-MPR
Giantin
μ1A(W408S)-GFP
CI-MPR
Giantin
E
μ1A(D174A)-GFP
CI-MPR
Giantin
μ1A(D174A)-GFP
CI-MPR
Giantin
F
WT cells + GFP
μ1A KO cells + GFP
μ1A KO cells + μ1A(WT)-GFP
μ1A KO cells + μ1A(W408S)-GFP
μ1A KO cells + μ1A(D174A)-GFP
Giatin co-localized with CI-MPR
(Mander's Coeeficient)
1.0
0.9
0.8
0.7
0.6
0.5
0.4
0.3
0.2
0.1
0.0
n.s.
* * *
* * *
* * *
n.s.

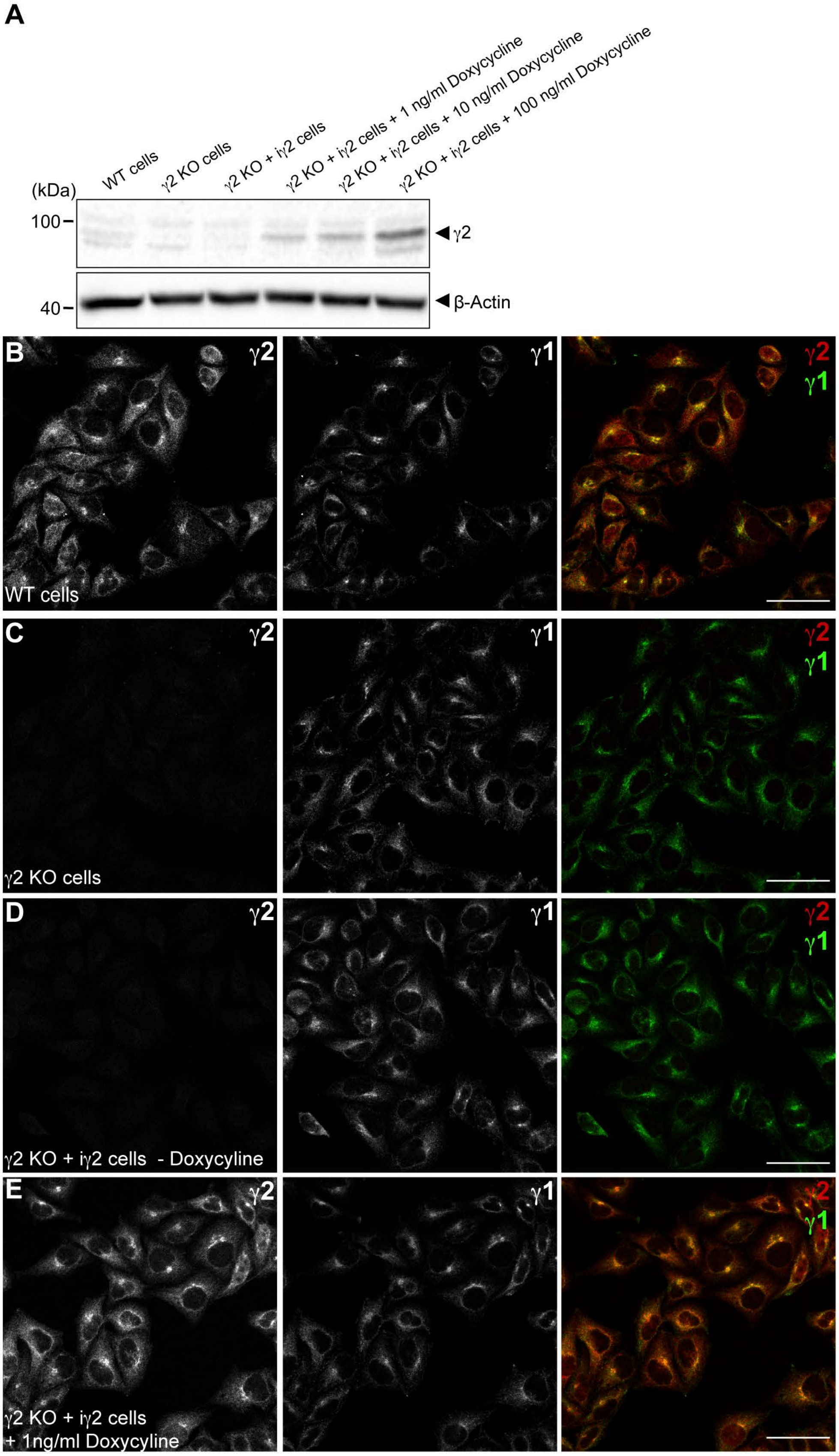
A
WT cells
γ2 KO cells
γ2 KO + iγ2 cells
γ2 KO + iγ2 cells + 1 ng/ml Doxycycline
γ2 KO + iγ2 cells + 10 ng/ml Doxycycline
γ2 KO + iγ2 cells + 100 ng/ml Doxycycline
(kDa)
100
40
γ2
β-Actin
B
γ2
γ1
WT cells
C
γ2 KO cells
D
γ2 KO + iγ2 cells - Doxycyline
E
γ2 KO + iγ2 cells
+ 1ng/ml Doxycyline

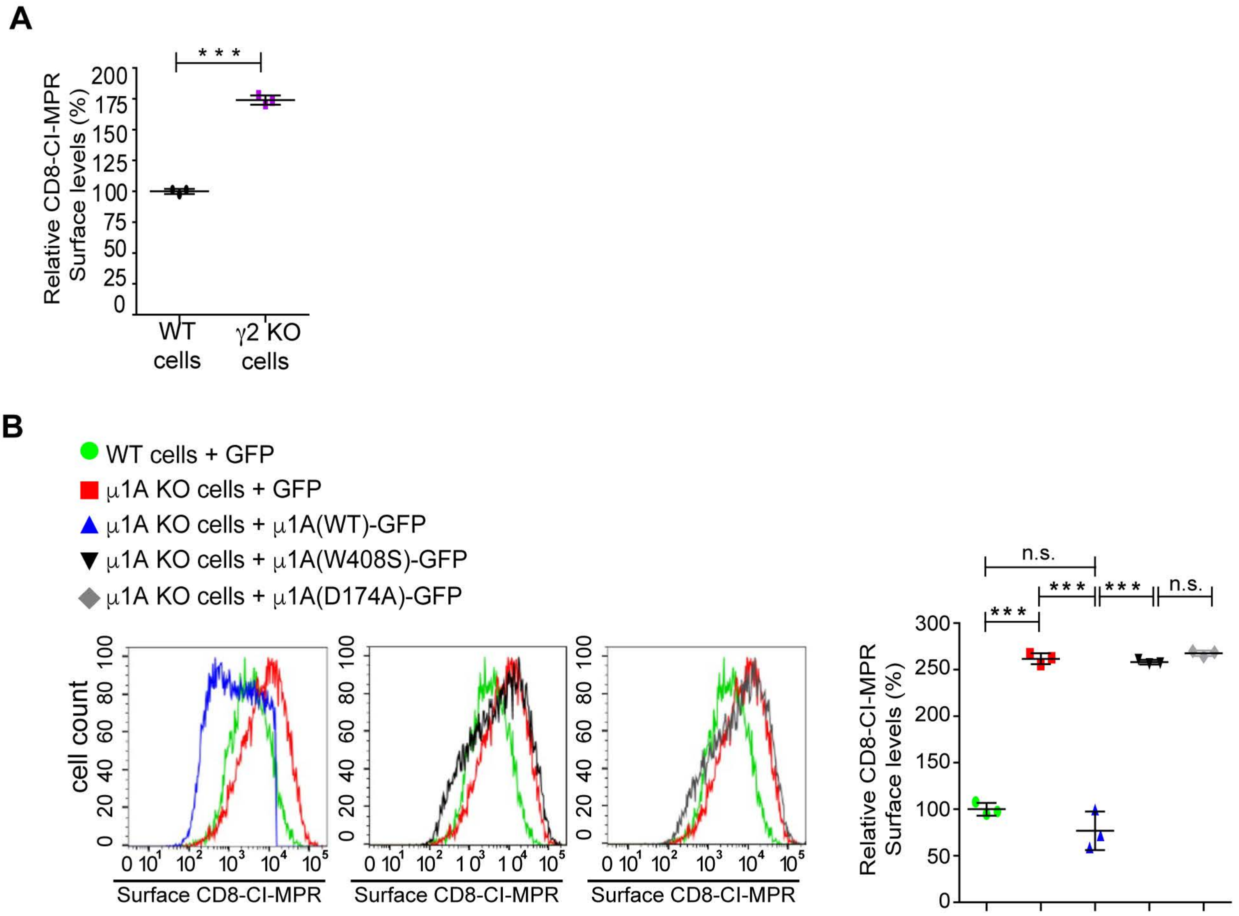
A
***
Relative CD8-CI-MPR Surface levels (%)
200
175
150
125
100
75
50
25
0
WT cells
γ2 KO cells
B
WT cells + GFP
μ1A KO cells + GFP
μ1A KO cells + μ1A(WT)-GFP
μ1A KO cells + μ1A(W408S)-GFP
μ1A KO cells + μ1A(D174A)-GFP
cell count
Surface CD8-CI-MPR
Surface CD8-CI-MPR
Surface CD8-CI-MPR
n.s.
***
***
***
n.s.
Relative CD8-CI-MPR Surface levels (%)
300
250
200
150
100
50
0

Fig. S5

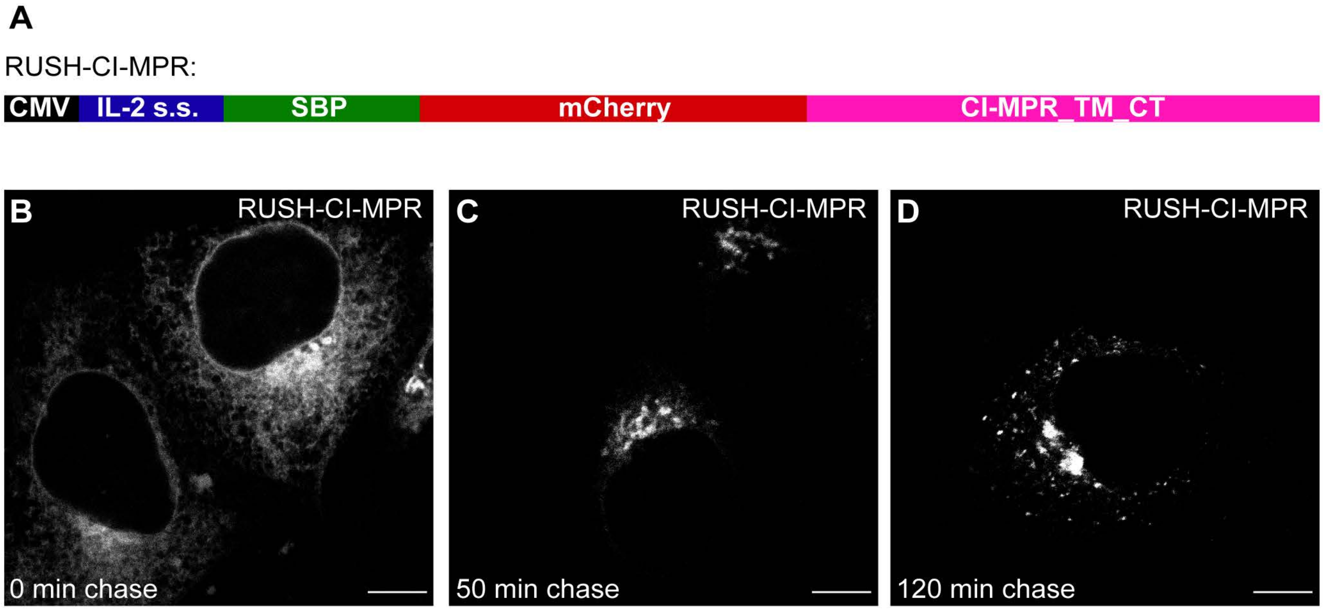
A
RUSH-CI-MPR:
CMV
IL-2 s.s.
SBP
mCherry
CI-MPR_TM_CT
B
RUSH-CI-MPR
0 min chase
C
RUSH-CI-MPR
50 min chase
D
RUSH-CI-MPR
120 min chase

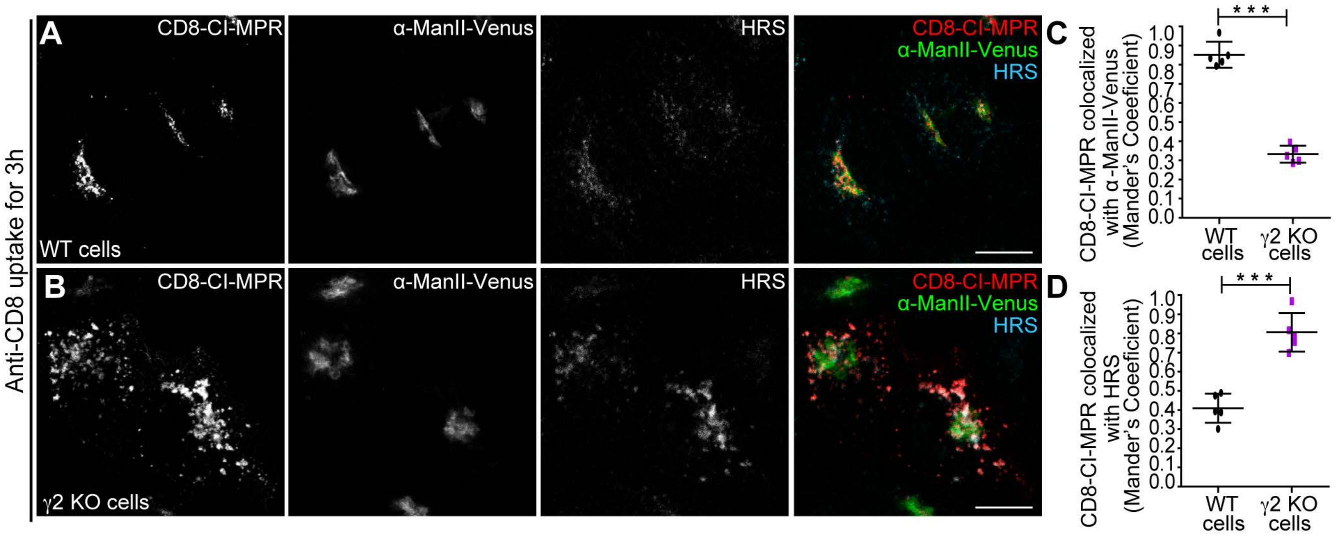
A
B
CD8-CI-MPR
α-ManII-Venus
HRS
CD8-CI-MPR
α-ManII-Venus
HRS
Anti-CD8 uptake for 3h
WT cells
γ2 KO cells
C
D
CD8-CI-MPR colocalized with α-ManII-Venus (Mander's Coeeficient)
CD8-CI-MPR colocalized with HRS (Mander's Coeeficient)
1.0
0.9
0.8
0.7
0.6
0.5
0.4
0.3
0.2
0.1
0.0
* * *
WT cells
γ2 KO cells

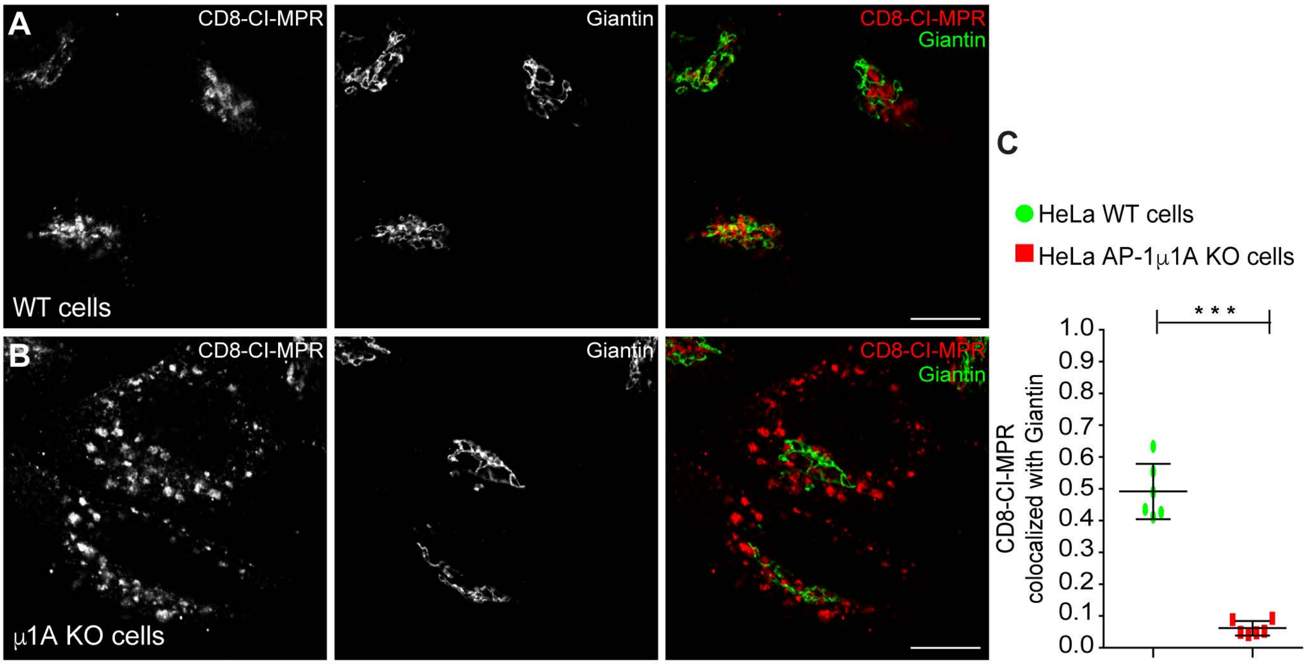
A
CD8-CI-MPR
Giantin
CD8-CI-MPR
Giantin
WT cells
B
CD8-CI-MPR
Giantin
CD8-CI-MPR
Giantin
μ1A KO cells
C
HeLa WT cells
HeLa AP-1μ1A KO cells
***
CD8-CI-MPR colocalized with Giantin
1.0
0.9
0.8
0.7
0.6
0.5
0.4
0.3
0.2
0.1
0.0
